# Disturbance Dynamics and Its Effects on Carbon in Human‐Impacted Mountain Forests in Northwestern Yunnan, China

**DOI:** 10.1002/ece3.72165

**Published:** 2025-09-14

**Authors:** Zhongqian Cheng, Tuomas Aakala, Chengjun Ji, Markku Larjavaara

**Affiliations:** ^1^ Institute of Ecology and Key Laboratory for Earth Surface Processes of the Ministry of Education, College of Urban and Environmental Sciences Peking University Beijing China; ^2^ Arthur Temple College of Forestry and Agriculture Stephen F. Austin State University Nacogdoches Texas USA; ^3^ School of Forest Sciences University of Eastern Finland Joensuu Finland; ^4^ Department of Forest Sciences University of Helsinki Helsinki Finland

**Keywords:** ecosystem carbon, fire, gap disturbance, human‐impacted mountain, logging, topography

## Abstract

Human activities alter disturbance regimes, influencing forest structure and ecosystem carbon. Identifying and quantifying natural and anthropogenic disturbances at fine spatial scales are critical to assessing the role of forests in climate change mitigation. This study investigated disturbance history and carbon storage in human‐impacted forests and open ecosystems at elevations of 2000–4200 m in northwestern Yunnan, China. We established 50 sampling plots along the four due orientations of a mountain peak. Using tree rings, fire scars, satellite imagery, official records, and interviews, we reconstructed historical disturbances and identified fires, logging events, landslides, and icy precipitation since the 1950s. We analyzed the impact of disturbance history and topography on ecosystem carbon storage, including pools in soil (0–30 cm), woody debris, and non‐woody and woody plants. Disturbances since the 1950s were largely driven by anthropogenic activities over time, along with climate and topography. Fires and logging were common near settlements in *Pinus yunnanensis* forests at lower elevations, while landslides primarily occurred in steep areas of *Abies georgei* forests and logged areas within broadleaf mixed forests. Icy precipitation was more frequent above 3500 m on the south and west slopes in *A. georgei* forests. Non‐forest areas at higher elevations had a mean ecosystem carbon (including soil carbon down to 30 cm) density of 146 Mg C ha^−1^, while forest areas averaged 270 Mg C ha^−1^. Fire negatively impacted soil, woody plants, and overall ecosystem carbon, whereas logging impacts were limited to woody plants and overall ecosystem carbon. Carbon storage in woody plants and total ecosystem carbon followed a hump‐shaped pattern with elevation, peaking near 3200 m. Our study links disturbance histories to spatial variation in carbon pools. This study helps improve carbon management and conserve biodiversity in human‐modified forests and presents a multi‐source approach that could be used in other human‐impacted forests.

## Graphical abstract

Identifying and quantifying natural and anthropogenic disturbances at fine spatial scales is critical to assess the role of forests in climate change mitigation. Using tree rings, fire scars, satellite imagery, official records, and interviews, we reconstructed historical disturbances and identified fires, logging events, landslides, and icy precipitation since the 1950s. Fire negatively impacted soil, woody plants, and overall ecosystem carbon, whereas logging impacts were limited to woody plants and overall ecosystem carbon.

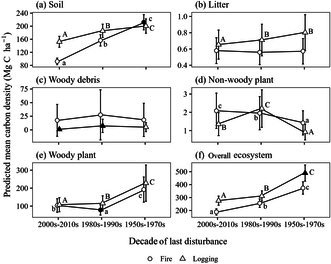

## 1 Introduction

Disturbances, both natural and anthropogenic, leave long‐lasting legacies on forest ecosystems (Running 2008). They drive key ecological processes, such as tree regeneration and growth, decomposition of woody debris, and the accumulation and release of soil carbon. Such processes regulate carbon dynamics (Brassard and Chen 2008; Fasth et al. 2011; Goetz et al. 2012; Turner and Seidl 2023). However, most forests worldwide are predominantly shaped by anthropogenic activities, though natural processes are fundamentally involved (Noble and Dirzo 1997; Vitousek et al. 1997). These activities include logging (Leverkus et al. 2021), land‐use change (Mantero et al. 2020), reforestation (Nolan et al. 2018), and fire suppression for intervening natural processes (Turner et al. 2003; Zhang et al. 2003). They can either intensify or mitigate the frequency and severity of natural disturbances. These changes further shift carbon storage patterns over time, interacting with natural carbon sequestration and release (Dye et al. 2024). Thus, addressing the impact of disturbances in human‐impacted forests is critical for enhancing our knowledge of forest carbon dynamics. Yet, such disturbance–carbon relationship remains inadequately understood.

Logging and land cover change directly influence forest dynamics. Their frequency and intensity can be intensified by increasing timber demands or halted by raising awareness of protection (Willson 2006; Goetz et al. 2012; Leverkus et al. 2021). Moreover, these anthropogenic activities are likely concentrated near populated areas or easily accessible locations (Aakala et al. 2023; Chen et al. 2024). In mountainous areas, for instance, topographically inaccessible regions are largely shaped by natural disturbances, while more accessible areas experience more frequent and intense human disturbances.

Human activities indirectly changed the characteristics of natural disturbances. For instance, anthropogenic activities can alter fire frequencies (Stambaugh and Guyette 2008; Wallenius 2011; Cerano‐Paredes et al. 2020; Margolis et al. 2022). Fire suppression commonly results in the accumulation of fuel, which can increase the severity of wildfires in the future (Turner et al. 2003). Such changes can further affect other disturbance regimes. For example, landslides are more likely to occur in areas that have experienced fire or deforestation (Zhang et al. 2003; Mantero et al. 2020; Leverkus et al. 2021).

Consequently, shifts in human interventions, driven by either socioeconomic needs or legislative changes, continuously alter the magnitude of natural and anthropogenic disturbances on forests over time. Periods of high economic demand and rapid social development often coincide with intensified human impact on forests, such as increased logging and land conversion (Xu and Ribot 2004). In contrast, in recent decades, as public awareness of environmental protection and conservation has grown, forests in many regions are beginning to recover from the impacts of past intensive logging (Zhang et al. 2003). Natural disturbance regimes may now follow altered trajectories shaped by historical land‐use changes and forest management practices.

Different disturbance agents have distinct impacts on various ecosystem carbon components. Fires, depending on their severity, typically cause an immediate reduction in aboveground biomass carbon while increasing the woody debris carbon (Turner et al. 2003; Goetz et al. 2012; Adinugroho et al. 2022). Their effects on soil and herbaceous plant carbon vary according to fire severity and ground cover (Kaye et al. 2010; Pellegrini et al. 2021). In contrast, logging primarily removes aboveground carbon and has minimal direct influence on soil carbon. However, its contribution to woody debris can vary depending on logging techniques and forest management policies. Some regulations require slash removal, while others promote deadwood retention for biodiversity (Ngo et al. 2013; Leverkus et al. 2021). Landslides result in the loss of both aboveground plants and soil carbon (Spooner et al. 2021). Storms, on the other hand, typically damage tree canopies and branches, leading to an increase in coarse woody debris carbon, though with less immediate impact on soil carbon (Fasth et al. 2011). Consequently, analyzing how individual disturbances affect each carbon component can fully explain the impact of disturbances on carbon dynamics.

Since 88% of global carbon turnover due to tree mortality is not able to be explained by stand‐replacing disturbances (Pugh et al. 2019), evaluating carbon dynamics caused by disturbances below the stand level is crucial. However, disturbances at such a fine scale are difficult to quantify by a single method. The methods applied to identify and quantify disturbances each have their limitations. For instance, tree basal scars can arise from various causes, such as fire, mechanical damage from logging, landslides, pest or pathogen infection, frost, or lightning (Molnar and McMinn 1960). This method can further provide timing and direction regarding disturbance events. But it may result in an incomplete record because it requires a proper intensity of disturbance and certain species to form those scars (Piha et al. 2013; Spooner et al. 2021). Satellite imagery offers another important data source, but systematic, large‐scale acquisition of Earth observation data only began in the 1970s (Cohen and Goward 2004), limiting the potential for longer‐term studies. Additionally, historical records and assessments of forest age and composition (Lorimer 1985) offer further sources of information on disturbance history, but their availability may be sporadic or relevant only in certain types of ecosystems and disturbances. Considering the limitations of each method, integrating multiple sources of information to detect sub‐stand level disturbances could be a possible solution; however, this is an approach rarely employed in a single study.

In this paper, we aim to evaluate the disturbance dynamics in human‐impacted forests by analyzing historical disturbances and their effects on above‐ and belowground carbon storage in forest ecosystems. Specifically, we sampled in a mountainous forest in northwestern Yunnan, China. This area represents the broader Jinsha River basin in northwestern Yunnan, as it shares similar forest types and a history of both natural and anthropogenic disturbances and provides a better representation than protected areas (Yang et al. 2016). Natural disturbances have been observed frequently, and the common disturbance agents in the area include fire, drought, landslide, flood, hail, or ice storms. Fire typically occurs in winter and spring when humid southwestern monsoonal winds are absent. Hail, a type of icy precipitation, had a frequency of once per year during 1961–2005 (Zhang et al. 2008). The susceptibility to landslides was due to the topography, lithological classification, geological structure, and precipitation pattern (Liu et al. 2023). On the other hand, human activities have been reported to have significantly altered the disturbance regimes and vegetation composition in southwestern Yunnan. In recent centuries, human‐caused fire during the dry season has become more predominant than lightning‐ignited ones (Xiao et al. 2017). Meanwhile, the recent decade of road construction makes this area more vulnerable to landslides (Liu et al. 2023). Vegetation reconstruction based on pollen records shows that modern vegetation represented only about 50% of the original natural assemblages, with a shift toward fire‐adapted *Pinus* spp. species (Li et al. 2019).

The objectives of this study were to (1) identify the timing of disturbances using tree‐ring records; (2) attribute these disturbances to specific agents from 1950 onward, using multiple data sources, including dendrochronologically dated fire scars, satellite images, historical documents, interviews, and field measurements of forest structure; and (3) determine the most relevant disturbances and analyze their influences on carbon storage in each ecosystem component, including soil, litter, woody debris, non‐woody and woody plants, and the overall ecosystem, while taking topography into consideration. We hypothesized that (1) topographic factors mediate the impact of disturbances on carbon storage, with elevation being the most important factor, and (2) different agents of disturbances (e.g., fire vs. logging) have distinct effects on carbon storage, and these effects may vary between ecosystem components.

## 2 Methods

### 2.1 Study Site

**FIGURE 1:**
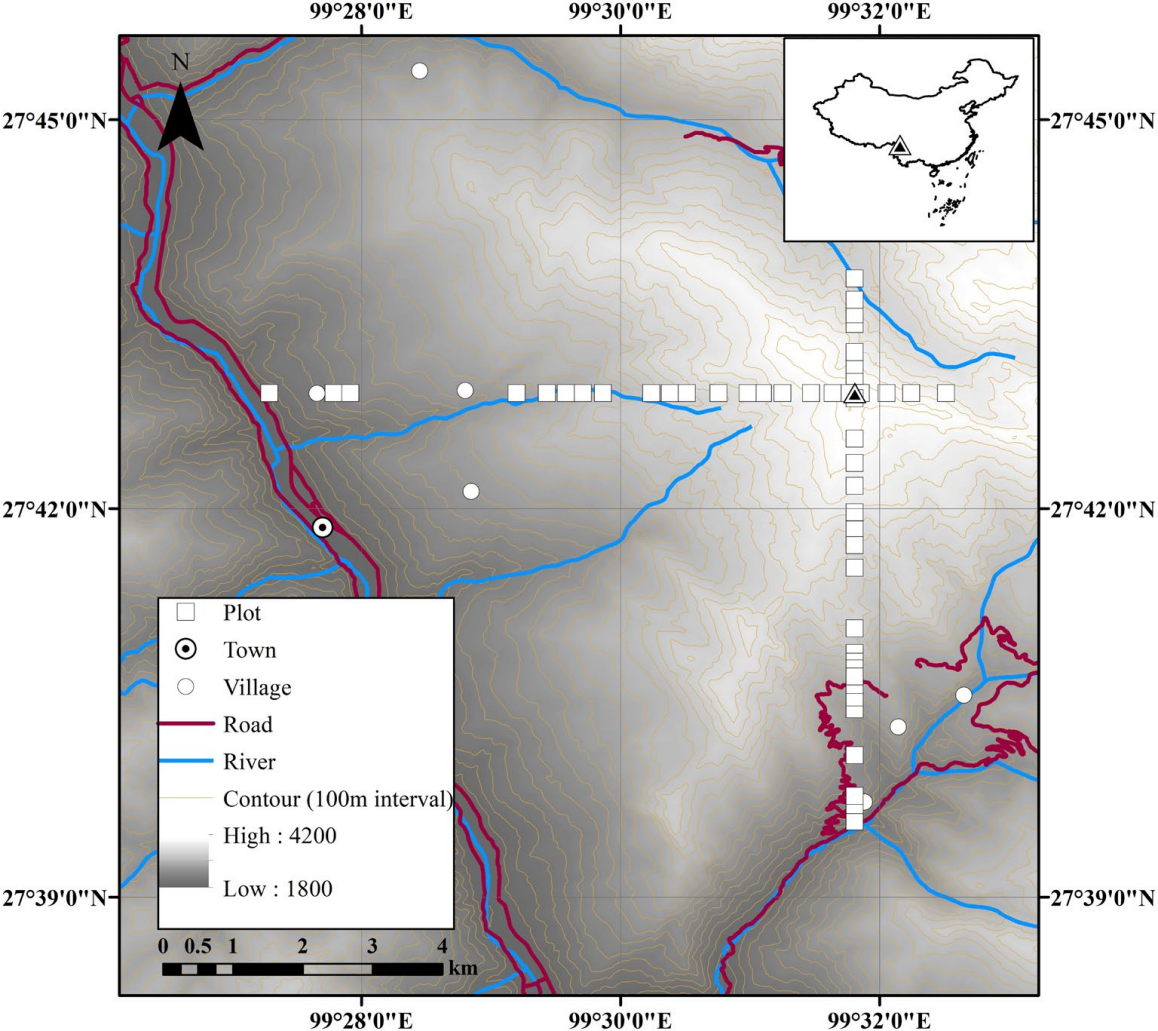
Location of study site and arrangement of plots.

Our study area was a mountain located on the eastern edge of the Himalayas (99.45°–99.54° E, 27.66°–27.73° N), in northwestern Yunnan province, China (Figure 1). The elevation ranges from 2000 m at the base to 4200 m at the summit. The mean annual air temperature recorded at the closest meteorological station (28.02° N; 99.73° E) at 3290 m was 5.2°C for the period 1958–2021. The mean annual precipitation was 702 mm, with > 90% of it occurring between April and October, derived from the National Meteorological Information Centre of China for the period 1958–2021 (https://www.cma.gov.cn/).

The vegetation types included arid valley shrubland below 2100 m on the west slope; young *Pinus yunnanensis* forest at 2400–3000 m on the west slope; mixed conifer‐broadleaf uneven‐aged forest dominated by *Pinus* spp. and *Pseudotsuga sinensis* at 2800–3300 m on both the west and south slopes; uneven‐aged broadleaf mixed forest dominated by *Acer* spp. below 2700 m on the south slope; mixed uneven‐aged forest dominated by *Acer* spp. and *Populus* spp. at 3400–3700 m; old *Quercus aquifolioides* forest over 100 years at 3300–3600 m on both the west and south slopes; uneven‐aged *Abies georgei* forest above 3800 m; and alpine shrubland and meadow above 3900 m on both slopes (Cheng et al. 2023).

Research or detailed documentation on the natural disturbances in this county is limited. However, like the general disturbance agents in northwestern Yunnan, the key disturbance agents that have been reported from the local forest administration and villagers include fire, landslide, flooding, hail, earthquake, drought, and pests. Among these, fire is considered the most critical concern for forest management planning. Fire prevention is a high priority; the local forest authorities invest substantial efforts in detection and suppression. No prescribed fire has been carried out in this area. Fires reported in recent decades were mostly caused by human carelessness during the dry season.

Logging, another main anthropogenic disturbance in this area, has been driven by the need for wood for construction, heating, and industrial purposes. Aligned with the forest management and logging practices at the national level, the regulations for logging have evolved significantly since the establishment of the People's Republic of China in 1949. In Southern China in general, logging intensified during the late 1950s–1960s and the subsequent late 1970s (Brown 2020), but it was largely halted with the 1998 Natural Forest Protection Program (Fang et al. 2018). As of 2018, 78% of forests in Yunnan were designated as community forests (National Forestry and Grassland Administration of China 2019), and in our study area, community‐based forest management has been in place since 1952 (Xu and Ribot 2004). Currently, timber harvesting requires permits from local authorities.

Species composition and structure within this system exhibit a legacy of these disturbances. For example, there were more *Quercus* spp. at the low to medium elevation historically but have been replaced with the fire‐dependent *P. yunnanensis* (Li et al. 2019). Shrub‐formed *Q. aquifolioides* was commonly found at low elevations or close to villages due to resprouting after fire or logging. After big trees were logged, pioneer species, such as *Acer* spp., *Rhododendron* spp., and *Populus* spp., proliferated at lower elevations.

### 2.2 Field Inventory

The mountain's highest point (transect cross in Figure 1) served as the primary reference. Circular inventory plots were systematically established along elevational gradients at 100 m intervals in four cardinal directions, starting from the peak and descending to the valley, excluding villages. A total of 50 plots were established: eight in the north aspect (3500–4200 m), four in the east aspect (3900–4200 m), 20 in the south aspect (2200–4200 m), and 18 in the west aspect (2000–4200 m). The length of each transect (in terms of elevation) varied because it either terminated at the valley or encountered an upslope. Density‐adapted circular plots, with radii of 5, 10, 15, and 20 m, were used depending on the number of trees within the 5 m radius, balancing sampling efficiency and accuracy (Jonsson et al. 2008).

In December 2021, trees with a diameter at breast height (DBH, 1.3 m) greater than 3 cm were counted and measured. The height of the first five trees of each species was measured using an ultrasound hypsometer (Haglöf Vertex Laser Geo, Haglöf Ltd., Sweden) or a 20‐m fishing rod, while the heights of the remaining trees were estimated based on the height–DBH relationship (Zhang et al. 2020). For shrubs, species, height, diameter at base, cover percentage, and number of individuals were recorded within a 2.5‐m radius subplot at the same center.

In November 2022, bryophytes, herbaceous vascular plants, woody debris, litter, and soil samples were collected for carbon analysis. Sampling began at the plot center, with three sub‐sampling points set 5 m from the center, each spaced 120° apart. These sub‐sampling plots were used as central points for collecting soil, litter (including woody debris < 2 cm), fine woody debris (FWD, 2–10 cm), and non‐woody plants. All litter, FWD, bryophytes, and herbaceous plants within a 0.5 × 0.5 m quadrat centered on each sub‐sampling plot were harvested. In all 150 subplots for the ground story, a total of 145, 25, and 147 samples for litter, FWD, and non‐woody plants were harvested.

Soil samples were collected to determine bulk density and organic carbon concentration. For bulk density, soil cores were taken from three layers: 0–10 cm, 10–20 cm, and 20–30 cm, using a 100 cm^3^ cylinder (Arrouays et al. 2018). For organic carbon concentration, a composite soil sample from the 0–30 cm depth was collected and stored in a plastic bag. In all 150 subplots for soil sampling, a total of 450 soil core samples and 150 composite soil samples were collected.

Coarse woody debris (CWD) with a diameter > 10 cm was measured within a 20‐m radius from the plot center. For logs (fallen deadwood), the length and diameter of both ends and the middle were measured. For snags (standing deadwood), height, DBH, and diameter at base were measured. Decomposition classes were assigned using a four‐level classification system (Table 1) as described by Zhu et al. (2017).

**TABLE 1:** Decay classes of coarse woody debris.

Decomposition class	Bark	Branch	Sapwood	Heartwood	Density (g/cm^3^)
I	Intact	Intact	Intact	Intact	0.3095
II	Bark loss, or easily removable bark	No criteria	Initial decay	Sound	0.2840
III	Bark lost	Large branch loss with stubs present	Decay or rotten	Decay	0.2020
IV	NA	NA	NA	Rotten	0.2020

In December 2021, all trees with a DBH over 10 cm were cored at 1.3 m height using 5.15‐mm increment borers (Haglöf Ltd., Sweden) to reconstruct disturbance history. Cores were stored in paper straws. Out of the 561 trees sampled in the plots, 459 were successfully cored. The remaining 102 trees, primarily *Quercus* spp., *Rhododendron* spp., and *Juglans* spp., could not be cored due to their dense wood or hollow trunks. Additionally, 21 of the cored trees yielded only partial cores because their stems were hollow in the center.

Fire scar samples were collected along the plot transects to reconstruct fire history (Figure S1 in Supporting Information). This kind of scar is formed after a tree heals from a fire. It is identified as a visible “cat face”‐like scar (Cerano‐Paredes et al. 2020). This is the result of cambial injury during surface fire events. In many cases, trees grow over these scars, allowing subsequent ring growth to partially or fully overtop the wound. Partial cross‐sections containing scars were obtained using a chainsaw. When trees were too large or regulations prohibited cutting, coring was used instead. Two increment cores were taken: one directly through the scar to identify the fire year and a second approximately 3 cm laterally from the scar, in undamaged wood, to reconstruct the tree's complete growth history (Cerano‐Paredes et al. 2020). All cores were stored in paper straws. Fire events identified in this way were assigned to adjacent plots based on the year of occurrence. A total of 15 fire scar samples were collected.

Cores and discs were air‐dried in a dark room at 25°C, with cores kept in paper straws during drying. Once dried, the cores were glued vertically onto mounts. All samples were sanded with progressively finer sandpaper until the tree rings became clear. The discs and cores were then scanned using an Epson Perfection V850 Pro scanner (Seiko Epson Corporation, Suwa, Japan) at 24‐bit color and 1200 dpi resolution. Tree‐ring boundaries and widths were identified and measured using CDendro 9.0 and CooRecorder 9.0 (Cybis Elektronik & Data AB, Sweden) with a resolution of 0.001 mm.

### 2.3 Biomass and Ecosystem Carbon Density

To estimate carbon storage in each ecosystem component, including soil, litter, woody debris, non‐woody and woody plants, and the overall ecosystem, we first measured tree and shrub sizes to determine their aboveground biomasses. Tree biomass was calculated using the pantropical aboveground biomass model (Chave et al. 2014). We selected this model after evaluating species‐ and genus‐level allometric equations using the dataset provided by Luo et al. (2019), which were either incomplete or less suitable for large trees and diverse species in our study area. Shrub biomass was estimated using species‐, genus‐, or biome‐ specific allometric equations (Table 2).

**TABLE 2:** Allometric equations for shrub used in this research.

Shrub species	Species allometric equation used	References
*Asparagua mgriacanthus*	*Symplocos ramosissima, Manglietia insignis, Camellia tsaii, Daphne papyracea, Ardisia crenata*	Zhang and Liu (2010)
*Berberis paraspecta*	*Berberis julianae*	Xie et al. (2018)
*Berberis yunnanensis*	*Berberis julianae*	Xie et al. (2018)
*Camellia crassipes*	*Camellia oleifera*	Xie et al. (2018)
*Camellia yunnanensis*	*Camellia oleifera*	Xie et al. (2018)
*Caragana erinacea*	*Caragana versicolor*	Xie et al. (2018)
*Caragana vitifolia*	*Caragana versicolor*	Xie et al. (2018)
*Coriaria nepalensis*	*Coriaria nepalensis*	Xie et al. (2018)
*Cotoneaster franchetii*	*Symplocos ramosissima, Manglietia insignis, Camellia tsaii, Daphne papyracea, Ardisia crenata*	Zhang and Liu (2010)
*Fargesia spathacea*	*Fargesia spathacea*	Xie et al. (2018)
*Hypericum uralum*	*Symplocos ramosissima, Manglietia insignis, Camellia tsaii, Daphne papyracea, Ardisia crenata*	Zhang and Liu (2010)
*Juniperus squamata*	*Juniperus squamata*	Xie et al. (2018)
*Mahonia longibracteata*	*Symplocos ramosissima, Manglietia insignis, Camellia tsaii, Daphne papyracea, Ardisia crenata*	Zhang and Liu (2010)
*Olea laxiflora*	*Symplocos ramosissima, Manglietia insignis, Camellia tsaii, Daphne papyracea, Ardisia crenata*	Zhang and Liu (2010)
*Quercus aquifolioides*	*Quercus monimotricha*	Xie et al. (2018)
*Rhododendron anthosphaerum*	*Rhododendron decorum; Rhododendron leucaspis*	Xie et al. (2018)
*Rhododendron beesianum*	*Rhododendron decorum; Rhododendron leucaspis*	Xie et al. (2018)
*Rhododendron calostrotum var. calciphilum*	*Rhododendron nivale*	Xie et al. (2018)
*Rhododendron erythrocalyx*	*Rhododendron decorum; Rhododendron leucaspis*	Xie et al. (2018)
*Rhododendron rupicola var. chryseum*	*Rhododendron nivale*	Xie et al. (2018)
*Rubus bonatianus*	*Rubus* spp.	Wang et al. (2012)
*Rubus rosifolius*	*Rubus* spp.	Wang et al. (2012)

To calculate tree belowground biomass, we used the root‐to‐shoot ratio from Luo et al. (2005) for trees older than 20 years, as well as for shrubs and vascular plants in the forested plots, shrublands, and alpine meadows, which provided estimates along subtropical to alpine gradients in the Tibetan Plateau. For trees younger than 20 years, we used the root‐to‐shoot ratio from Luo et al. (2012), which stratified biomass allocation by forest age classes (Table 3). For shrub and herbaceous vascular plants in non‐forested plots, we applied the Chinese shrubland and grassland root‐to‐shoot ratios of 0.73 and 6.00, respectively (Wang et al. 2014). Litter, FWD, and non‐woody plant biomass were calculated with dried samples at 70°C until a constant weight was reached.

**TABLE 3:** Root‐to‐shoot ratio used in this research.

Plants group from the references	Root‐to‐shoot ratio	Plot/species used in our study
Older trees/stands (≥ 20 years) Luo et al. (2005)		
Alpine shrub–meadow of *Rhododendron* spp.—*Kobresia* spp.	0.9279	S3900; S4000; S4100; S4200; W4000; W4100; W4200
Other subtropical pines and conifers Luo et al. (2012)	0.2020	W2400; W2500; W2800
Mixed forest of *Pinus densata* and *Quercus* spp. evergreen trees	0.3439	S2500; S3000; S3100; S3200; S3400; W3200
Timberline forest of *Abies georgei*	0.1455	N3900; N4000; N4100; E3900; E4000; E4100; E4200; S3700; S3800; W3700; W3800; W3900
Evergreen broadleaved forest	0.4083	N4200
Evergreen–deciduous broadleaved forest	0.2728	N3500; N3600; N3700; S2200; S2400; S2700; S2800; S2900; S3300; W2900; W3000; W3100; W3300; W3400; W3500; W3600
Mixed forest of spruce–fir and deciduous trees	0.0739	N3800; S3500; S3600
Young tree/stands (< 20 years) Luo et al. (2012)		
*Abies* spp., *Picea* spp., and *Sabina* spp.	0.217	*Abies* sp. sapling in N3700, N3900, E4000, S3600, S3700; *Abies* sp.in W3500
Other subtropical pines and conifers	0.266	*Pinus yunnanensis* sapling in S3100, W2400, W2500; *Tsuga* sp. sapling in S3400, W3000; *Pinus armandii* in S2900; *Pinus armandii* in W3000; *Pinus yunnanensis* in S2400; *Pseudotsuga sinensis* in W3100
*Alnus* spp., *Betula* spp., and *Populus* spp.	0.254	*Populus* sp. sapling in W3100
*Quercus* spp. and other temperate deciduous broadleaved forests	0.306	*Quercus* spp. sapling in W3100; *Quercus acutissima* in S2500; *Quercus aquifolioides* in S2500, S3400; *Quercus dentata* in S2400
Other subtropical deciduous broadleaved forests	0.397	*Acer* spp. sapling in S3600, S3700; *Sorbus* sp. sapling in S2700; *Cinnamomum* spp. sapling in S2800; *Padus* sp. sapling in W3000; *Terminalia franchetii* in W3700
Other evergreen broadleaved forests	0.249	*Photinia* sp. in S2200; *Rhododendron anthosphaerum* in S2500
Subtropical coniferous–broadleaved mixed forest	0.266	*Tilia* sp. sapling in N3800; *Prunus* sp. sapling in E3900; *Crataegus* sp. sapling in W3900

To calculate CWD biomass, we first calculated CWD volume, assuming a frustum shape. Measuring the top diameter of snags was challenging. Therefore, we used the relationship between diameter at base and breast height and current snag height to estimate the height when snags were alive, thereby simulating the diameter at the top. Subsequently, we calculated the biomass of CWD by multiplying its volume by the density of the four decay classes (Table 1).

To estimate snag height when alive, we used our own plot‐level data of the living trees to fit the DBH–height relationship, following the form described in Zhang et al. (2020). This DBH–height relationship used the nonlinear least squares model to estimate parameters *a* and *b*. The estimation results were *a* = 1.72434 and *b* = 0.55810 (*R* ^2^ = 0.55), respectively.

Therefore, the relationship between the height and DBH of living trees in our research area was expressed as follows:

$$H={\mathrm{DBH}}^{1.72434}+0.58810$$

where *H* is height in m and DBH is the diameter at breast height of standing deadwood in cm.

If the predicted height exceeded the measured height in the field, we used the current height to estimate the diameter at the top.

To estimate the top diameter of standing deadwood, we assumed a linear taper of the tree stem from the base to the top. We used the DBH, diameter at base, deadwood current height, and the estimated height when the dead trees were alive to calculate the diameter at the top:

$$D_{\mathrm{top}}=\left(1-\frac{H-1.3}{H_{e}-1.3}\right)\times \mathrm{DBH}$$

where *H* is the current height of standing deadwood in m, *H*_*e*_ is the estimated height when the tree was alive, and DBH is the diameter at breast height of standing deadwood in cm.

Alternatively, if the estimated height and measured deadwood height were the same:

$$D_{\mathrm{top}}=\frac{\mathrm{DBH}\times H+1.3\times D_{\text{base}}-D_{\text{base}}\times H}{1.3}$$

where *D*_base_ is the base diameter in cm, and the rest are as defined above.

For soil bulk density analysis, samples were dried at 105°C until a constant weight was achieved. Stones and roots within each soil core were weighed separately. Bulk density was calculated as the ratio of the oven‐dried mass of soil to the internal volume of the cylindrical soil corer (100 cm^3^) used for sample collection.

For soil organic carbon concentration analysis, samples were air‐dried until a constant weight was achieved. Roots and stones were manually removed before sieving the soil (< 2 mm) (Ngo et al. 2013). Soil organic carbon concentrations were then determined using the K_2_Cr_2_O_7_ titration method (Nelson and Sommers 1996).

Ecosystem carbon estimation followed the Intergovernmental Panel on Climate Change (IPCC) guideline for National Greenhouse Gas Inventories for forest land and grassland (IPCC 2006, 2019). Woody tissue carbon fractions were obtained from Doraisami et al. (2022). The data set was filtered using the following criteria: biomes—subtropical, regions—Asia, for all living plants. We used the mean (0.47) of this filtered result. Carbon fractions for non‐woody plants and litter were taken from IPCC (2006) as 0.47 and 0.37, respectively.

Total carbon density (*C*_Total_, Mg·C ha^−1^) was calculated as follows:

$$C_{\text{Total}}=C_{\mathrm{AB}}+C_{\mathrm{BB}}+C_{\mathrm{NW}}+C_{L}+C_{\mathrm{WD}}+C_{\mathrm{SO}}$$

where *C*_*AB*_ is the carbon stored in aboveground biomass of woody plants, C_*BB*_ is the carbon stored in belowground biomass of woody plants, C_*L*_ represents the carbon stored in litter, C_*NW*_ is the carbon stored in non‐woody plants, C_*WD*_ is the carbon stored in woody debris, and C_*SO*_ represents the carbon stored in soil (0–30 cm).

Aboveground carbon density (*C*_*AB*_, Mg·C·ha^−1^) was calculated as follows:

$$C_{\mathrm{AB}}=\sum_{i}B_{{\mathrm{AB}}_{i}}\times {\mathrm{CF}}_{i}$$

where *B*_*ABi*_ is the species‐specific aboveground biomass in each plot (Mg·ha^−1^), and *CF*_*i*_ is the carbon fraction of dry matter.

Belowground carbon density (*C*_*BB*_, Mg·ha^−1^) was calculated as follows:

$$C_{\mathrm{BB}}=\sum_{i}B_{{\mathrm{AB}}_{i}}\times R_{i}\times {\mathrm{CF}}_{i}$$

where *B*_*ABi*_ is the species‐specific aboveground biomass in each plot (Mg·ha^−1^), *R*_*i*_ is the species‐specific ratio of belowground biomass to aboveground biomass, and *CF*_*i*_ is the carbon fraction of dry matter.

Non‐woody plants carbon density (*C*_*NW*_, Mg·C ha^−1^) was calculated as follows:

$$C_{\mathrm{NW}}=B_{\mathrm{NW}}\times \mathrm{CF}$$

where *B*_*NW*_ is the biomass of non‐woody plants, and *CF* is the carbon fraction of dry matter.

Litter carbon density (CL, Mg·C·ha^−1^) was calculated as follows:

$$C_{L}=B_{L}\times \mathrm{CF}$$

where *B*_*L*_ is litter biomass and *CF* is the carbon fraction of dry matter.

Woody debris carbon density (*C*_*WD*_, Mg·C ha^−1^) was calculated as follows:

$$C_{\mathrm{WD}}=B_{\mathrm{FWD}}\times \mathrm{CF}+V_{\mathrm{CWD}}\times \rho \times \mathrm{CF}$$

where *B*_FWD_ is the biomass of fine woody debris, *V*_CWD_ is the volume of coarse woody debris, *ρ* is the density by various decay classes, and *CF* is the carbon fraction of dry matter.

Soil carbon density up to 30 cm in depth (*C*_*SO*_, Mg·ha^−1^) was calculated as follows (Tadiello et al. 2022):

$$C_{\mathrm{SO}}=\mathrm{OC}\times \mathrm{LT}\times \mathrm{BD}\times \left(1-\mathrm{RF}\right)\times 10^{4}$$

where OC is organic carbon concentration (%), LT is layer thickness (cm), BD is soil bulk density (Mg·m^−3^), and RF is rock fragment content fraction.

### 2.4 Timing of Disturbances

Regarding the fire scar samples, each fire‐scarred tree had only one visible scar, indicating a single fire event. Therefore, the year of fire occurrence was determined by counting the rings formed after the scar. Since the fire scars were sampled along transects between plots to avoid damaging the plots for further remeasurement, potential fire events were assigned to both adjacent plots once the year was identified.

**FIGURE 2:**
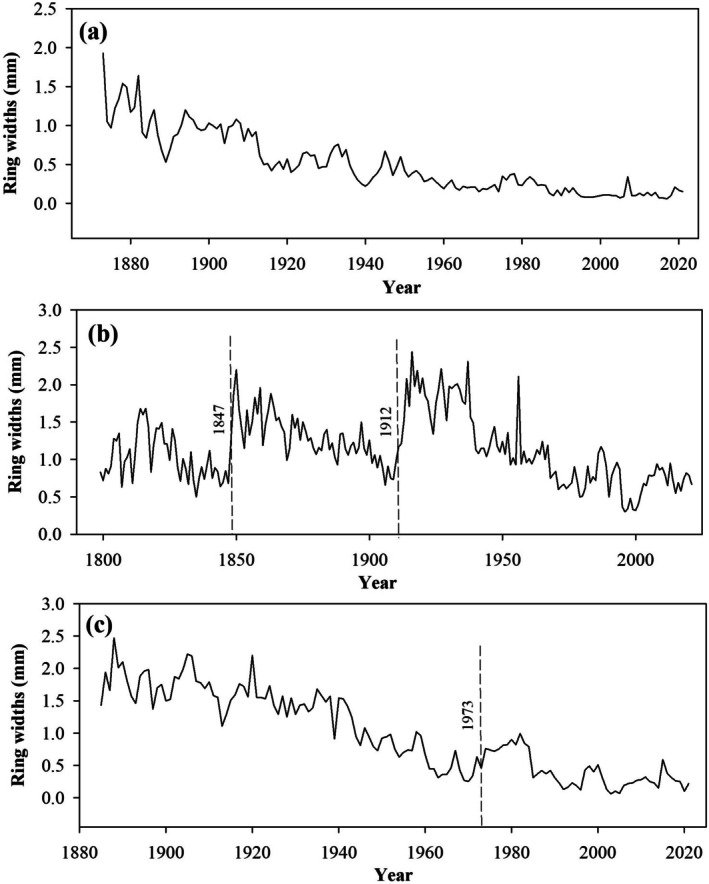
Examples of gap‐origin trees, growth release, and pseudo‐growth release. (a) Gap‐origin tree; (b) A non‐gap‐origin tree with two disturbances in 1947 and 1912; (c) Gap‐origin tree with one pseudo‐growth release in 1973.

Disturbances other than fire, which may not leave visible marks on trees, can still be detected through abrupt, sustained increases in radial growth of surviving trees. Such growth surges occur due to increased resource availability following the death of an overtopping or large neighboring tree (Lorimer and Frelich 1989). To identify such growth releases, we used the running mean method (Nowacki and Abrams 1997) with a 10‐year moving window, applying a 50% growth increase as the release criterion (see growth pattern example in Figure 2b). This analysis was conducted using the TRADER package (Altman et al. 2014) in RStudio (Positteam 2023). All identified releases were visually checked to ensure they were abrupt, sustained, and not the results of year‐to‐year growth variation due to favorable environmental conditions (Figure 2c for an example of such a “pseudo‐release”). Gap‐origin trees (Figure 2a) are also indicators of disturbances, characterized by high initial growth followed by a gradual decline in growth rate (Lorimer and Frelich 1989). This pattern occurs when trees regenerate after canopy openings, with rapid early growth that slows as the canopy closes around the gap‐origin tree (Lorimer et al. 1988). We firstly screened all the samples and filtered those with such a pattern, then we established a threshold based on the mean tree‐ring width during the first 15 years for each species (Aakala et al. 2011). While species‐specific growth rates can vary, trees regenerating under closed‐canopy conditions consistently exhibited narrow early ring widths due to light limitations. To be more efficient, we thus applied a universal threshold of 1.49 mm average radial width, and any initial growth rate exceeding this value was classified as a gap‐origin event.

Disturbance timing, reconstructed from the growth release and gap‐origin analyses, was combined to create a disturbance chronology. For each decade, the disturbance intensity was reported as the proportion of trees exhibiting release signals or gap‐origin traits out of the total number of trees alive during that decade. Tree establishment and mortality dates were determined from ring‐width series. Since this method employed a 10‐year moving window, growth releases occurring in the most recent decade were not included.

In addition to disturbance, natural mortality may also create canopy gaps. However, forests in this area were uneven‐aged. Natural mortality in such forests typically involves single trees dying, creating small canopy gaps or no gaps that may not be detected by our method. In contrast, disturbances tend to remove groups of trees, creating larger gaps. These larger disturbances are more likely to alter light availability, potentially influencing the growth patterns of neighboring living trees. Therefore, natural mortality was excluded from our method.

### 2.5 Disturbances Agents Since 1950

While tree‐ring records indicate that disturbance events occurred as early as the late 1600s (see Section 3.1 Disturbance chronologies), our analysis of disturbance agents was restricted to the period since 1950. This selection was due to (1) the earliest fire events detected through fire scars occurring in 1933; (2) those local villagers recalling the earliest disturbance agents from the 1950s; and (3) this being the time when most historical documents that we collected became reliable.

#### 2.5.1 Yearbooks

To identify the disturbance agents and timings, we collected data from various sources. In addition to dating fire by fire scars, we also reviewed a series of Yunnan Disaster Prevention Yearbooks from 1991 to 2018 for obtaining records and documentation of past disturbances in the study area (Editorial Committee of Yunnan Disaster Reduction Yearbook 1997, 1999, 2000, 2002, 2004, 2006, 2008, 2010, 2012, 2014, 2016, 2018, 2020). These 13 yearbooks presented information on disturbance agents, duration, and severity. We gathered data on various disaster types using keywords such as drought, earthquake, fire, icy precipitation, landslide, snow, low temperature, storm, warm temperature, and wind. The Chinese translations used in the yearbook searches for the keywords were drought “干旱/旱灾”, earthquake “地震”, fire “火灾/火情”, icy “冰雹/冻雨/冰暴”, landslide “泥石流/山体滑坡/塌方”, snow “雪灾”, low temperature “低温/霜冻”, storm “暴雨/暴风雨”, warm temperature “高温”, and wind “风灾”. We were able to identify fire occurrences in our study area. For other disturbances, the search was expanded to adjacent municipalities to investigate whether potential events occurred close to our research mountain but were not reported in our remote study area. Meanwhile, these disturbances could occur simultaneously with the events reported in the yearbooks.

#### 2.5.2 Satellite‐Based Maps

To complement the disturbance history, 30‐m resolution satellite‐based maps (Hansen et al. 2013; Tyukavina et al. 2022) were analyzed to detect forest loss events caused by both fire and non‐fire disturbances. The Global Forest Change data set, spanning from 2000 to 2021, was used to identify the annual forest area that is potentially lost to fire (Tyukavina et al. 2022) and non‐fire (Hansen et al. 2013) causes. The algorithm defined loss as a removal of woody plants exceeding 5 m in height, caused by fires or other factors. These sources of satellite‐based maps could help us in determining the spatial extent and severity of forest fires, deforestation, or land cover changes. Spectral algorithms of the annual fire/non‐fire forest loss addressed disturbances that cause most of the land cover change. Consequently, we assumed that the adjacent areas potentially experienced the same disturbances but at a lower intensity, which escaped detection by the spectral indices. To account for this and to broaden the possible explanations for the agents behind the disturbances detected from tree rings, we partitioned the mountain into eight orientations, represented as compass angles from 0° to 360°. Disturbances occurring between 315° and 45° were categorized within the north transect. Similarly, disturbances in the range of 45° to 135° were assigned to the east transect, those between 135° and 225° to the south transect, and disturbances spanning from 225° to 315° to the west transect.

#### 2.5.3 Interviews

In our study, semi‐structured interviews were conducted with individuals from the closest villages to the research area (Adeoye‐Olatunde and Olenik 2007). The villages were Chedatang, Tangzhu, Kangsegang, Jiaotang, Jiaogang, Naoren, and Wujing (villages can be seen in Figure 1). A total of 20 villagers, comprising 13 men and seven women, aged between 45 and 86 years, were interviewed, along with two staff members from the local forest farm. These individuals were selected based on their familiarity with the region and their potential knowledge concerning historical disturbances. During the semi‐structured interviews, we asked questions related to natural disturbances and their occurrence, to human use of the land and forest, and how these may have changed through time, and we encouraged the interviewees to respond in more depth:

1. What was and is harvested wood used for?
2. Which species were and are harvested, and how many cubic meters can be harvested for one household?
3. When was mass logging/harvesting conducted, and at which location? We specifically asked about the Great Leap period and about the periods prior to and after the natural forest protection project.
4. Do you remember whether any natural hazards, including but not limited to landslides, droughts, storms, earthquakes, and floods, have occurred?
5. Do you remember whether any forest fires have occurred?
6. Do you remember whether any pest or pathogen outbreaks have occurred that caused significant tree mortality?
7. Do you plant any trees, or has the local authority organized tree plantings? We specifically asked about the period before and after Grain for Green, a nationwide forestry project launched in 1999 aiming to convert cultivated land into forests or grassland (Delang and Yuan 2015).
8. Since when have people started raising yaks and cattle?

#### 2.5.4 Additional Information

In addition, several indicators were used to pinpoint potential disturbances. Local communities have historically used fire for agricultural purposes (e.g., prescribed burning), for heating, and to protect livestock. Sometimes these fires spread unintentionally, causing forest fires. In the 1950s, for instance, local villagers reported using fire to deter carnivores, and corresponding fire scars were found in the south and west aspects. A significant fire event in 1999 was documented in the yearbooks and confirmed by several villages, with evidence of the fire's spread from the base to the top of the tree line in the south aspect. In the late 1980s, road expansion projects were initiated to facilitate timber transport, particularly from villages to riversides. Two logging trails, heavily used during the 1980s, were also identified at the base of the north aspect in a gully and along the west aspect between 2900 and 3500 m in elevation. This information was assigned as a cue for logging activities.

We integrated the results from fire scars, yearbooks, satellite data, and interviews (see Figure S2 in Supporting Information for methods) and matched disturbance agents to observe growth releases for each plot and decade from the 1950s to the 2010s. Within this time frame, we focus on disturbances that lead to tree mortality, including fires, landslides, icy precipitation, and logging. We excluded drought and wind events, although they were frequently recorded in the yearbooks and reported in the interviews. These two agents generally do not produce the localized, gap‐scale mortality patterns required for reliable detection via growth release methods in the study area. Wind disturbances either result in broad‐scale blowdowns or interact with landslides or ice precipitation, but the latter two agents are most likely to have caused direct and abrupt tree mortality. We were unable to identify specific types of landslides or frozen precipitation events responsible for tree mortality. Therefore, we used the umbrella term “landslide” to include debris flows, rockslides, and avalanches. Similarly, the term “icy precipitation” was used to refer to ice storms, hailstorms, freezing rain, or other frozen precipitation events.

Pine caterpillar (*Dendrolinus houi*) and shoot beetle ( *Tomicus piniperda* ) have been reported to be causing damage to *P. yunnanensis* (Yu et al. 2018). However, due to the proactive pest prevention measures taken by the local forest farm, such as applying pesticides and using larva traps, these two types of pests are not common in the study area. Therefore, pests and diseases were not considered disturbance agents.

A disturbance agent was assigned to a given plot and decade only when two or more independent sources supported the attribution, and the spatial and temporal data sources aligned with the growth release or gap‐origin signals. Where this criterion was not met, the event was excluded from agent‐specific analysis. Therefore, such criteria led to the exclusion of approximately 25% of the growth releases for which no clear cause could be identified from the available data. Non‐fire forest loss between 2000 and 2021, detected through satellite imagery, was attributed to logging, icy precipitation, or landslides based on the specific location. When multiple disturbance agents were recorded in a plot, a hierarchy of priority was followed: fire, then landslide, followed by icy precipitation, and finally logging.

### 2.6 Topographic Factors

The topographic wetness index (TWI) was computed using System for Automated Geoscientific Analyses (SAGA) GIS software, following the methodology outlined by Conrad et al. (2015). TWI was calculated as follows:

$$\mathrm{TWI}=\ln\left(\frac{\frac{\mathrm{TCA}}{\mathrm{FW}}}{\tan\left(S\right)}\right)$$

where TCA represents the total catchment area, FW represents flow width, and *S* represents slope degree.

For our study, we began with an ALOS Digital Elevation Model (DEM) with a 30‐m resolution. To optimize the DEM for hydrological analysis, we determined slope degree by following the nine‐parameter second‐order polynomial method introduced by Zevenbergen and Thorne (2006). We then applied the depression‐filling technique outlined by Planchon and Darboux (2002). For flow routing, we adopted the multiple flow direction algorithm proposed by Quinn et al. (2006). This algorithm, which distributes flow to all downslope cells, includes adjustments that consider the maximum flow accumulation in adjacent cells characterized by modest slopes.

Topographic position index (TPI) was calculated based on Weiss (2011) as follows:

$$\mathrm{TPI}=\mathrm{int}\;\mathrm{DEM}-\text{focalmean}\;\left(\mathrm{DEM},\text{annulus},\text{irad},\text{orad}\right)+0.5$$

where irad is the inner radius of each cell, and orad is the outer radius of each cell.

### 2.7 Influences of Disturbance and Topography on Carbon Density

To determine the key environmental factors influencing carbon storage, we conducted a factor analysis of mixed data (Table 4). The analysis included both continuous and discrete variables. Continuous variables at the plot level encompassed topographic factors such as elevation, north–south orientation of aspect (cosine of azimuth degree), east–west orientation of aspect (sine of azimuth degree), slope, TWI, and TPI. Disturbance factors, including the decade of the last fire, logging, landslide, or icy precipitation, were treated as discrete variables to avoid fitting an inaccurate temporal trend. The decades of the last disturbance were classified into four categories: “no recent,” “1950s–1970s,” “1980s–1990s,” and “2000s–2010s.” These breakpoints were chosen to reflect the large‐scale commercial logging conducted in the 1980s–1990s and the logging ban implemented in 1998. We found that elevation and TWI were the two most relevant topographic factors. While our identification of fires and logging as the most widespread disturbances across the study area was based on the synthesis of multiple independent data sources, the factor analysis served to validate their importance. Landslides and icy precipitation, on the other hand, occurred more locally.

**TABLE 4:** The results of factor analysis of mixed data of environmental variables, including the contribution of each variable to each dimension, eigenvalues of each dimension, and overall explained variation.

	Dimension 1	Dimension 2	Dimension 3	Dimension 4	Dimension 5
Elevation	19.80	0.14	4.49	5.43	1.00
AspectNS	9.64	2.41	2.22	13.90	< 0.01
AspectEW	10.38	11.35	7.26	3.17	0.51
Slope	0.08	0.10	23.96	1.32	9.41
TWI	14.67	18.28	3.71	0.02	0.01
TPI	12.03	5.17	5.74	2.13	0.02
No recent fire	4.25	7.79	0.02	1.14	0.81
Fire in 1950s–1970s	1.85	5.76	2.68	9.93	1.74
Fire in 1980s–1990s	1.32	0.31	6.11	3.99	22.77
Fire in 2000s–2010s	1.66	5.61	2.37	17.14	7.65
No resent logging	2.92	0.15	7.28	2.17	0.79
Logging in 1950s–1970s	0.21	6.85	0.58	0.02	20.57
Logging in 1980s–1990s	2.29	1.97	0.66	10.66	1.81
Logging in 2000s–2010s	0.28	0.73	0.95	4.18	11.72
No recent landslide	0.56	2.54	0.56	0.09	0.76
Landslide in 1950s–1970s	2.06	0.11	0.02	0.45	3.36
Landslide in 1980s–1990s	8.13	4.73	4.25	0.70	0.17
Landslide in 2000s–2010s	7.00	23.73	0.05	0.33	6.63
No recent icy precipitation	0.10	0.28	1.72	3.13	0.62
Icy precipitation in 1980s–1990s	0.09	1.44	0.02	11.75	0.75
Icy precipitation in 2000s–2010s	0.57	0.53	16.34	8.34	8.81
Eigenvalues	3.15	2.05	1.91	1.72	1.35
Explained variation %	18.51	12.05	11.23	10.09	7.94
Accumulated explained variation %	18.51	30.56	41.80	51.89	59.82

To further assess the effects of disturbances and topography on ecosystem carbon, we employed a generalized additive model (GAM). The model evaluated the impact of the decade of the last fire and last logging, as well as elevation and TWI, on carbon stocks in woody plants, herbaceous vascular plants, woody debris, soil, and the overall ecosystem. For model simplicity and to avoid multicollinearity, we excluded disturbance frequency from the analysis, particularly because sites with frequent disturbances tended to have experienced recent disturbances. Meanwhile, initial analyses showed that the timing of the last disturbance had a stronger and more direct effect on carbon storage than frequency.

The effect of elevation was modeled using a two‐knot cubic regression spline to capture typical plant patterns along elevational gradients (Rowe and Lidgard 2009; Acharya et al. 2011), allowing for either linear or unimodal responses. The smoothing parameters were estimated using generalized cross‐validation (GCV), and the model was fitted using a Gaussian distribution with log transformation. We used the k‐index to assess the basic dimensions for smooth variables, and residual diagnostics are provided in Figure S3 (Supporting Information for methods).

All analyses and visualizations were carried out in R (RCoreTeam 2024). To compare predicted mean carbon densities across different decades of last disturbance, we performed an analysis of variance (ANOVA) followed by Tukey's honest significant difference (HSD) test for multiple comparisons. The factor analysis was performed using the *factoextra* (Kassambara and Mundt 2020) and *FactoMineR* (Lê et al. 2008) packages. The GAM analysis was conducted using the *mgcv* package (Wood 2017).

## 3 Results

### 3.1 Disturbance Chronologies

**FIGURE 3:**
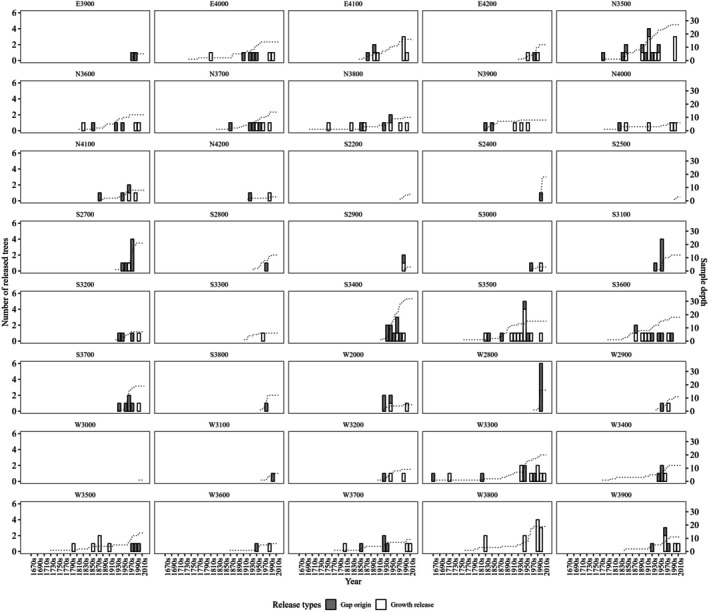
Disturbance chronologies on a decadal scale of each plot. The number of release trees separated by release type is shown as a bar with corresponding values shown on the left *y*‐axis. Sample depth (number of trees) is shown as a dashed line with corresponding values shown on the right *y*‐axis.

Of the 459 trees cored, 173 (37.4%) exhibited at least one growth release, and 39 trees recorded multiple growth releases (Figure 3). Additionally, 108 gap‐origin trees were identified (see Figure 3). At the plot level, 37 out of the 42 forest plots showed evidence of disturbance, either through growth releases or gap‐origin trees. Three plots, located on the south aspect at 2200 m (S2200) and 2500 m (S2500) and on the west aspect at 3000 m (W3000), showed no evidence of disturbance from either growth releases or gap‐origin trees. Two additional plots (W2400 and W2500) contained only trees that did not meet the 10 cm DBH size threshold for sampling.

**FIGURE 4:**
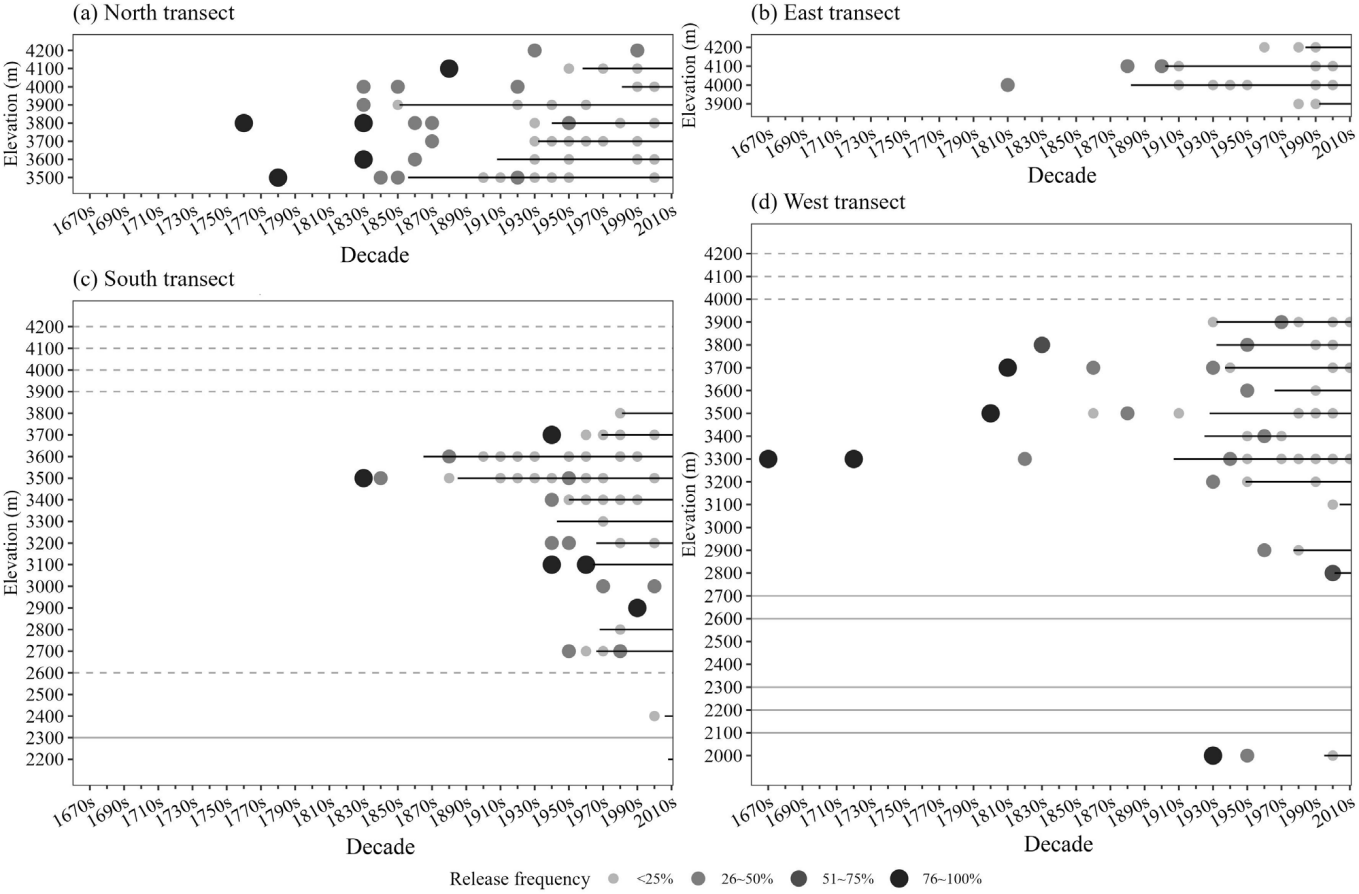
Release frequency at the decadal scale along elevational gradients in the north aspect (a), east aspect (b), south aspect (c), and west aspect (d). Each point represents the release frequency, with varying point sizes and colors corresponding to different frequency ranges: Less than 25% (smallest, light gray), 26%–50% (medium gray), 51%–75% (dark gray), and 76%–100% (largest, black). Black solid lines indicate more than five trees released during that decade; gray solid lines indicate no plot at the corresponding elevation; gray dashed lines indicate non‐forest plots.

We combined growth releases and gap‐origin trees into a decadal resolution and defined plot‐level disturbance chronology as any decade in which trees in the plot showed signs of either growth release or gap origin. We identified a total of 161 disturbance events. The timing of the earliest detected disturbances varied by aspect, ranging from 1670 to 1830 (Figure 4). The average decadal disturbance frequencies (defined as the number of trees with growth release or gap origin divided by the total number of trees at that decade) were 0.30 (±0.04 SE), 0.19 (±0.03), 0.26 (±0.04), and 0.31 (±0.04) for the north, east, south, and west aspects, respectively.

In terms of disturbances since 1950, in the north aspect, four out of eight plots showed disturbances during the 2000s (Figure 4a), while five plots recorded disturbances in both the 1950s and 1990s. In the east aspect, all four plots recorded disturbances during the 1990s, although disturbance frequencies remained below 25% (Figure 4b). In the south aspect, disturbances were consistently observed from the 1950s to the 1990s, affecting plots from 2700 m up to around 3600–3700 m (Figure 4c). In the west aspect, disturbances primarily occurred between 3200 m and 3900 m, near the upper tree line, from the 1950s onward (Figure 4d).

### 3.2 Disturbance Agents Since 1950

**FIGURE 5:**
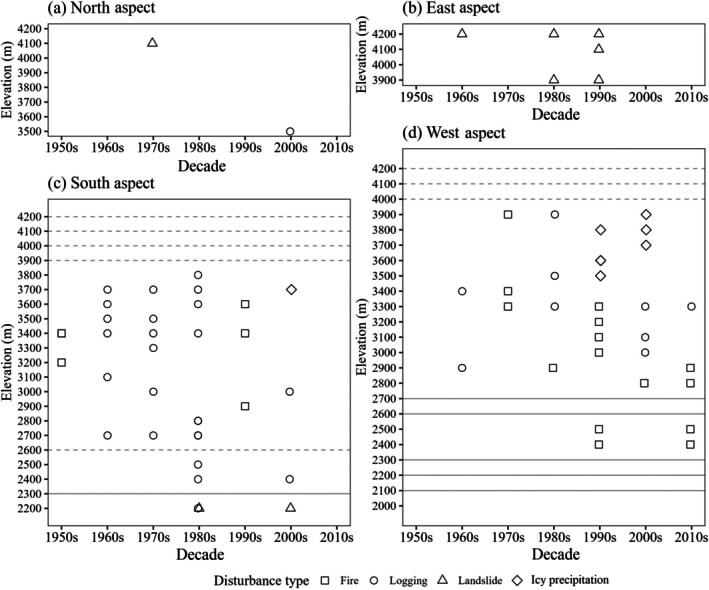
Disturbance agents at the decadal scale since the 1950s along elevational gradients in the north aspect (a), east aspect (b), south aspect (c), and west aspect (d). Gray solid lines indicate no plot at the corresponding elevation; gray dashed lines indicate non‐forest plots.

By comparing the disturbance chronology from tree‐ring data with historical records, we estimated the occurrence of fire, logging, landslides, and icy precipitation since 1950 (Figure 5a–d). Divided by aspect, the south and west aspects exhibited a higher frequency of fire and logging disturbances, while both the north and east aspects were primarily marked by landslides, which is consistent with the presence of gap‐origin trees.

Of the disturbances detected in the north aspect, N4100 (plot ID defined as transect and elevation) in the 1970s was attributed to a landslide, while the event at N3500 in the 2000s was due to logging (Figure 5a). In the east aspect, disturbances were detected at E4200 in the 1960s; at E4200 and E900 in the 1980s; and at E4200, E4100, and E3900 in the 1990s, all of which were caused by landslides (Figure 5b). In the south aspect, disturbances in the 1950s and 1990s were attributed to fires, while disturbances at S3700 in the 2000s were caused by icy precipitation. Disturbances from the 1960s to the 1980s were mainly due to logging, with logging activities also detected at S2400 and S3000 in the 2000s (Figure 5c). In the west aspect, disturbances in the 1950s, 1970s, 1990s, 2000s, and 2010s were caused by fire, as indicated by fire scars. Disturbances above 3500 m in the 1990s were caused by icy precipitation, while those in the 1960s, 1980s, 2000s, and 2010s were attributed to logging (Figure 5d).

Additionally, we inferred the disturbance history of plots S2200, S2500, and W3000 directly from historical documents, where no release events or gap‐origin trees were recorded. These plots experienced stand‐replacing disturbances. For instance, S2200 suffered a landslide in the 1980s, while trees below S2500 were cut during the same period. Fire scar samples indicated fires in W3300 and in most plots below it in the 1990s. Interviews, yearbooks, and fire scars confirmed fires at S2400 and S2500 in the late 1990s. Based on satellite‐based maps and disturbance chronology, logging was determined to have occurred in W3000 during the 1990s.

We connected several mountain huts used by herdsmen to nearby fires and logging based on the conclusions of the interviews. One hut was located in the valley at the foot of the mountain between the east and north aspects (N3500 and E3900), while two others were positioned between W3500 and W3700, and two more were above S3800. Wood around these huts was harvested for construction, firewood, and carnivore‐deterring fires. The local fire intended to deter carnivores occurred about 30 years ago.

### 3.3 Carbon Density Distribution

**FIGURE 6:**
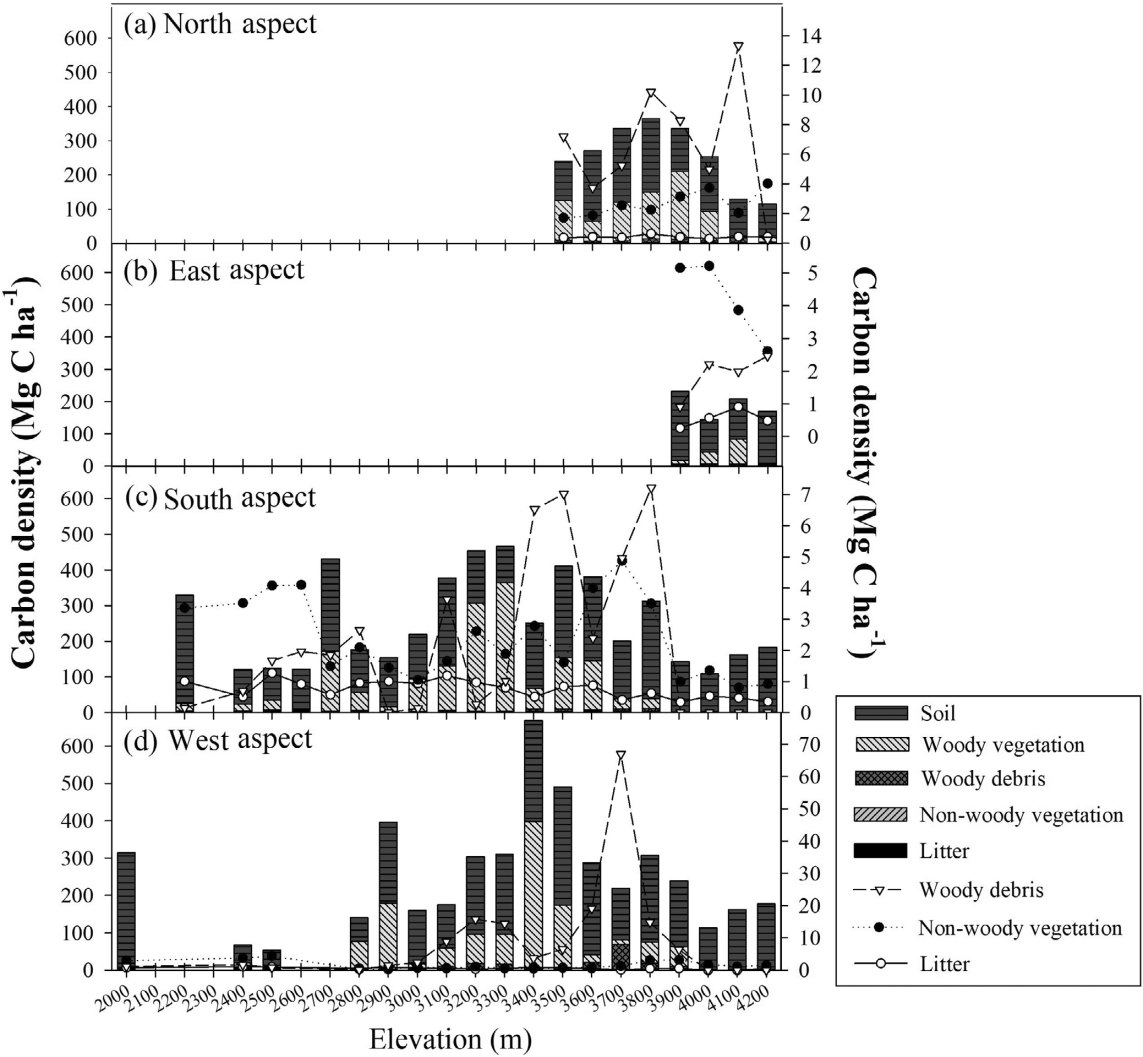
Carbon density of soil, woody plants, woody debris, non‐woody plants, and litter along elevational gradients in the north aspect (a), east aspect (b), south aspect (c), and west aspect (d). Each bar represents the carbon density of a specific ecosystem component. The cumulative value displayed represents the overall ecosystem carbon density, with corresponding values indicated on the left *y*‐axis. In cases where the carbon density values of woody debris, non‐woody plants, and litter are relatively small and difficult to distinguish within the stacked bars, their values are additionally presented as points, referenced on the right *y*‐axis.

Across all forest plots, ecosystem carbon density exhibited considerable variation, ranging from 52.9 to 668.2 Mg C ha^−1^, with an average of 269.9 Mg C ha^−1^. The majority of this carbon was stored in the soil, contributing 64.5% to the total, while woody plants accounted for 32.0%. The carbon density within woody plants alone ranged from 1.9 to 392.6 Mg C ha^−1^, with an average of 86.3 Mg C ha^−1^. In contrast, soil carbon density was slightly more uniform, ranging from 40.8 to 316.8 Mg C ha^−1^, and averaging 174.2 Mg C ha^−1^. Non‐woody plants contributed relatively little, with carbon densities ranging from 0.2 to 5.2 Mg C ha^−1^, averaging 2.4 Mg C ha^−1^. Litter and woody debris were even smaller contributors, with litter carbon density varying from 0.1 to 1.3 Mg C ha^−1^, averaging 0.7 Mg C ha^−1^, and woody debris ranging from 0 to 67.0 Mg C ha^−1^, averaging 6.3 Mg C ha^−1^ (Figure 6a–d).

In non‐forest plots, the overall ecosystem carbon density was lower, ranging from 108.7 to 182.7 Mg C ha^−1^, with an average of 146.4 Mg C ha^−1^. Soil carbon was the dominant contributor in these areas, accounting for 98.5% of the total carbon stored, ranging from 106.8 to 181.4 Mg C ha^−1^, and averaging 144.2 Mg C ha^−1^. Woody plant carbon density in non‐forest plots was minimal, ranging from less than 0.01 to 0.07 Mg C ha^−1^, with an average of just 0.01 Mg C ha^−1^. Non‐woody plant carbon was also low, ranging from 0.8 to 4.1 Mg C ha^−1^, with an average of 1.6 Mg C ha^−1^. Litter and woody debris made only minor contributions, with litter carbon density ranging from 0 to 0.9 Mg C ha^−1^ (averaging 0.4 Mg C ha^−1^) and woody debris ranging from 0 to 2.0 Mg C ha^−1^ (averaging 0.3 Mg C ha^−1^; Figure 6a–d).

### 3.4 Influences of Disturbances and Topography on Forest Ecosystem Carbon

**FIGURE 7:**
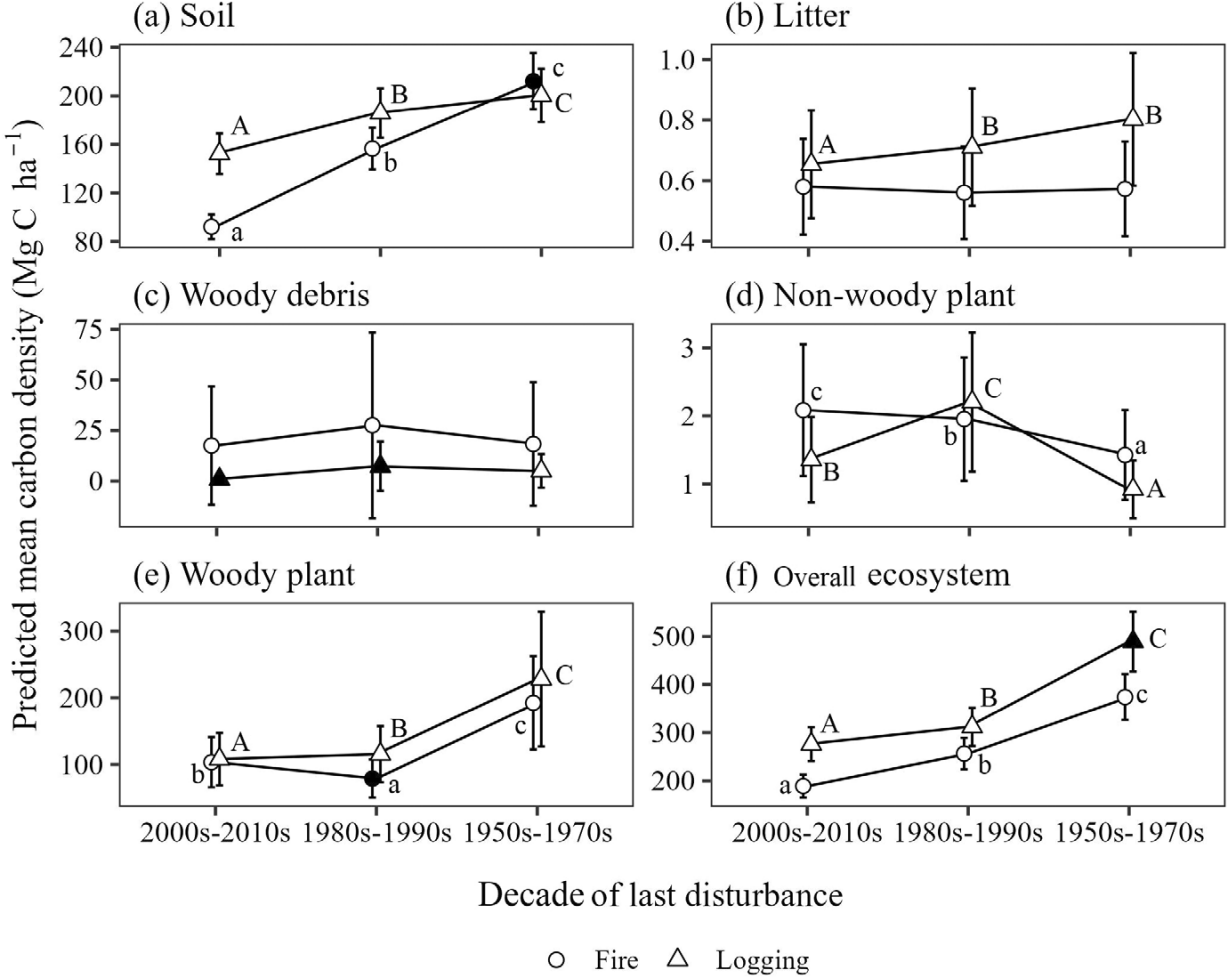
Predicted mean carbon densities of soil (a), litter (b), woody debris (c), non‐woody plants (d), woody plants (e), and overall ecosystem (f) in the study area, fitted using generalized additive models for different decades since the last fire or logging event. Significant coefficients between decades since the last fire or logging events are presented as black points, while non‐significant ones are shown as gray points. Error bars represent the standard deviation of predicted values. Lowercase letters indicate significant differences among decades since the last fire, while uppercase letters indicate significant differences among decades since the last logging.

We identified significant differences in soil carbon density between fires occurring in the 1950s–1970s and the absence of recent fire, as well as in woody plant carbon density between fires in the 1980s–1990s and no recent fire (Table 5). Soil carbon density showed a significant increase from plots disturbed in the 2000s–2010s to plots disturbed in the 1950s–1970s, indicating substantial carbon accumulation over time. Similarly, woody plants and whole ecosystem carbon densities exhibited significant increases, reflecting a recovery and enhancement of carbon storage in these components post‐disturbance. In contrast, litter and woody debris carbon densities showed slight, non‐significant increases, remaining relatively stable. Non‐woody plant carbon density experienced a significant decrease, suggesting that fire might have a long‐term suppressive effect on this component (Figure 7). The post‐fire carbon recovery rate of the whole ecosystem (defined as the slope of the predicted whole ecosystem carbon by year) in this study was approximately 3.76 Mg C ha^−1^ year^−1^, indicating a steady pace of recovery in overall carbon storage following fires.

**TABLE 5:** Generalized additive model statistics for carbon components in ecosystems in relation to fire disturbances, logging disturbances, and topographic factors. Fire and logging are treated as categorical variables, with the reference category being the absence of recent fire or logging. The values shown are standardized coefficients. Models were fitted using data from *n* = 42 sampling plots.

		Soil	Litter	Woody debris	Non‐woody plants	Woody plants	Overall ecosystem
Intercept	Estimate	4.848	−0.266	−2.220	1.103	4.428	5.352
	SE	0.163	0.169	0.950	0.228	0.530	0.175
	*p*	**< 0.001**	0.125	0.026	**< 0.001**	**< 0.001**	**< 0.001**
Fire in 1950s–1970s	Estimate	0.275	−0.550	−0.025	−0.227	0.062	0.159
	SE	0.130	0.173	0.250	0.231	0.213	0.124
	*p*	**0.043**	0.731	0.920	0.332	0.773	0.208
Fire in 1980s–1990s	Estimate	0.118	−0.045	0.296	−0.096	−0.998	−0.196
	SE	0.161	0.153	0.588	0.270	0.454	0.175
	*p*	0.468	0.772	0.618	0.726	**0.035**	0.273
Fire in 2000s–2010s	Estimate	−0.541	−0.026	−0.382	0.077	−0.515	−0.467
	SE	0.349	0.197	6.575	0.327	0.437	0.269
	*p*	0.131	0.882	0.954	0.814	0.247	0.092
Logging in 1950s–1970s	Estimate	0.223	0.275	−1.327	−0.623	0.426	0.425
	SE	0.179	0.164	0.686	0.591	0.219	0.153
	*p*	0.221	0.103	0.062	0.300	0.060	**0.009**
Logging in 1980s–1990s	Estimate	0.147	0.158	−0.862	0.273	−0.218	0.051
	SE	0.133	0.143	0.361	0.200	0.331	0.138
	*p*	0.276	0.278	**0.023**	0.182	0.515	0.712
Logging in 2000s–2010s	Estimate	−0.139	0.033	−2.902	−0.236	0.609	−0.230
	SE	0.198	0.176	0.520	0.349	0.390	0.200
	*p*	0.487	0.851	**< 0.001**	0.504	0.128	0.259
TWI	Estimate	0.044	−0.047	0.589	−0.041	−0.018	0.046
	SE	0.022	0.027	0.077	0.034	0.099	0.028
	*p*	0.054	0.090	**< 0.001**	0.243	0.854	0.116
s(Elevation)	edf	1.000	1.000	1.922	1.880	1.931	1.898
	ref.df	1.000	1.000	1.994	1.986	1.995	1.990
	*F*	0.001	15.730	8.403	4.196	5.926	3.696
	*p*	0.982	**< 0.001**	**0.002**	**0.032**	**0.005**	**0.034**
Adjust *R* ^2^		0.279	0.293	0.862	0.337	0.600	0.496
Deviance explained		0.419	0.431	0.881	0.480	0.685	0.605

*Note:* Values in bold indicate statistical significance at the *p* < 0.05 level.

Abbreviations: edf, effective degrees of freedom; ref.df, reference degrees of freedom; s, smoothed variables; SE, standard error; TWI, topographic wetness index.

**FIGURE 8:**
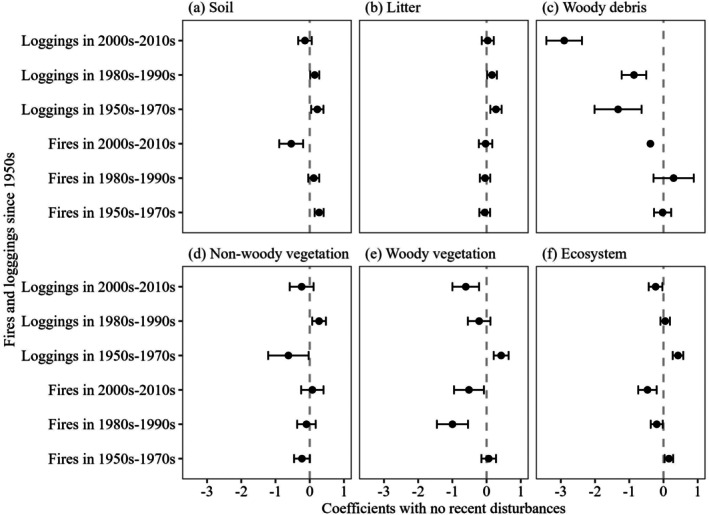
Standardized coefficients of soil (a), litter (b), woody debris (c), non‐woody plants (d), woody plants (e), and ecosystem (f) carbon density between no recent disturbances with fires and loggings in the 1950s–1970s, 1980s–1990s, and 2000s–2010s, respectively. Error bars of woody debris between no recent disturbances with fires in the 2000s–2010s are not shown due to their large value that exceeds the range.

**FIGURE 9:**
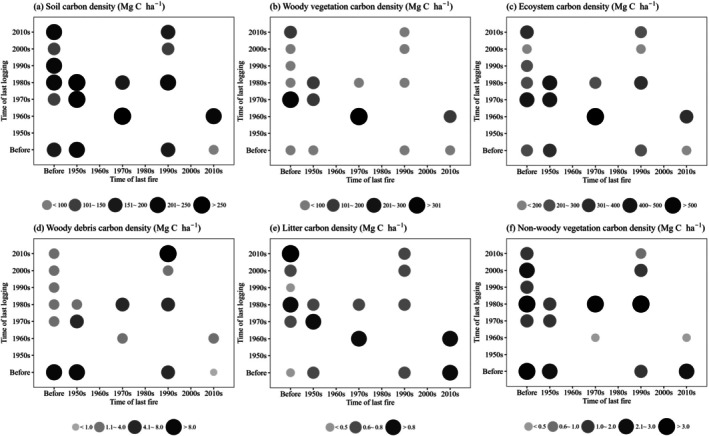
Carbon density difference of ecosystems and their components in each period considering fire and logging.

Significant differences in woody plant carbon density were observed between plots that experienced logging in any decade since the 1950s and those without recent logging (Table 5). Soil carbon density was significantly higher in areas last logged during the 2000s–2010s compared to those logged in the 1950s–1970s, following a similar but less pronounced trend as observed with fire‐affected areas. In comparison to areas without recent logging activity, the decade in which the last logging occurred had a relatively minor effect on soil properties (Figure 8). Litter carbon density increased in areas last logged during the 2000s–2010s compared to those logged in the 1980s–1990s. Woody plant and overall ecosystem carbon densities significantly increased, with logged areas showing higher values compared to those affected by fire in recent decades. Non‐woody plant carbon density was the highest in the area last logged during the 1980s–1990s. Woody debris carbon density remains stable and non‐significant across the decades, akin to the pattern observed following fire. The post‐logging carbon recovery rate of the whole ecosystem was approximately 4.26 Mg C ha^−1^ year^−1^, reflecting a faster rate of carbon accumulation and recovery in ecosystems impacted by logging compared to those affected by fire. Mean carbon densities for each period, considering both fire and logging, are shown in Figure 9.

**FIGURE 10:**
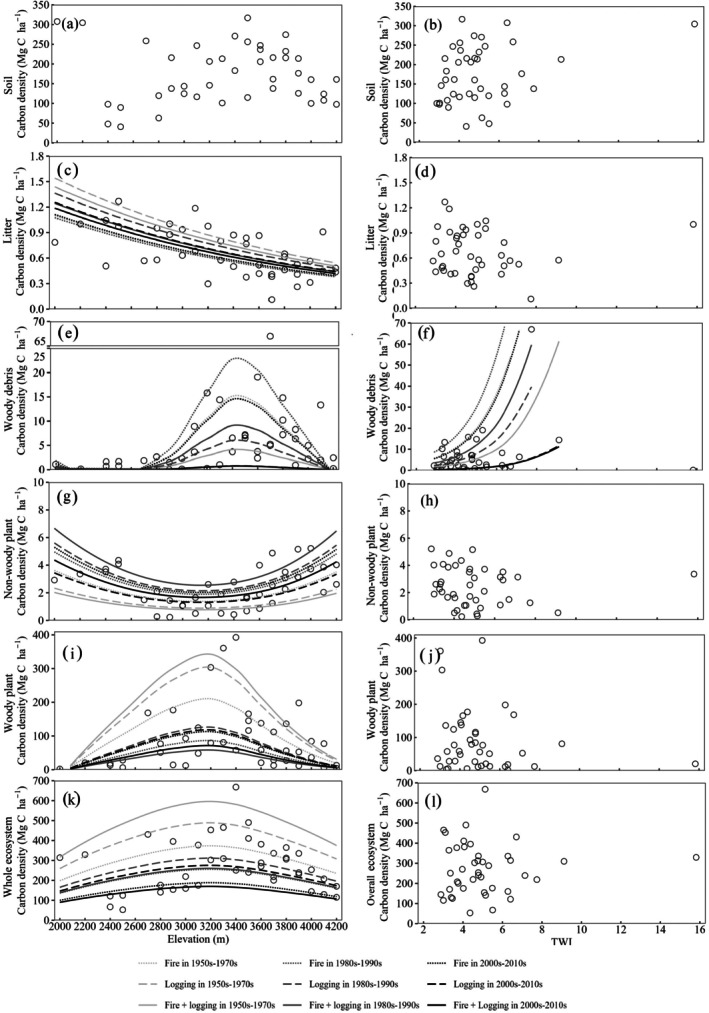
Carbon density distribution of soil (a, b), litter (c, d), woody debris (e, f), non‐woody plants (g, h), woody plants (i, j), and overall ecosystem (k, l) along elevational gradients and the topographic wetness index, respectively. Smoothing lines for each decade of disturbance affecting the response variables were fitted using generalized additive models utilizing median values for other variables.

Regarding topographic attributes, elevation affected litter, woody debris, non‐woody plants, woody plants, and overall ecosystem carbon densities, but not soil carbon density. Litter carbon density displayed a decreasing trend with increasing elevation, while the other four components displayed a unimodal response to elevation. Woody debris, woody plants, and overall ecosystem carbon densities peaked at moderate elevations, whereas non‐woody plant carbon density was lowest at intermediate elevations. A high TWI was associated with high woody debris carbon density (*p* < 0.001, Figure 10).

## 4 Discussion

Since the 1950s, anthropogenic activities have shaped disturbance regimes, including the occurrence of fires, landslides, and the intensity of logging, in this mountainous forest ecosystem of northwestern Yunnan. We also found these disturbances were partly influenced by evolving policies and economic development, indicating socioeconomic changes in the region. In addition to human activities, topographic factors, particularly elevation, had a notable impact on the distribution and severity of natural disturbances, including fires, landslides, and icy precipitation, in this study area.

The two most prevalent disturbances, fires and logging, had distinct and differing impacts on the primary carbon components of the ecosystem: soil and woody plant carbon stocks. Fires led to reductions in soil carbon, woody plant biomass, and overall ecosystem carbon, while logging primarily affected woody plant carbon and, consequently, overall ecosystem carbon. Elevation, acting as a mediator of climatic variation, shaped the woody plant and overall ecosystem carbon dynamics regardless of disturbance history. Together, our results underscore the significant impact of human interventions on forest dynamics, both directly by altering disturbance regimes and indirectly by affecting carbon storage through their influence on these disturbances.

### 4.1 Disturbance History

Fires in the study area were influenced by both climate and human activities. Consistent with previous research, warmer and drier conditions on south‐ and west‐facing slopes, as well as lower elevations, facilitated fire ignition and spread (Rogeau and Armstrong 2017; Wolf et al. 2021). Regulations shaped the occurrence of fire since the 1950s, a pattern also addressed in northern Europe (Aakala et al. 2023) and the western United States (Turner et al. 2003), where specific policies influenced fire dynamics over time. The lack of fire control regulations between the 1950s and 1970s allowed fires to ignite and spread across various mountain areas, where they occurred across a larger elevational range—between 3200 and 3400 m in the south aspect and between 3300 and 3900 m in the west aspect, as indicated by our disturbance agents' results (Figure 5). Herding activities could be one of the causes of fire (Zhang et al. 2003). With the implementation of more effective fire suppression policies from the 1980s onward (Zhang et al. 2003), most fires were limited to accidental ignitions near villages. However, we did not find that intensified fire suppression efforts could lead to the outbreak of large‐scale wildfires in this area, a phenomenon potentially linked to similar practices observed in the western United States, as mentioned by Turner et al. (2003). This was largely due to villagers regularly collecting large quantities of litter, particularly pine needles, for use as bedding for domestic animals and as fertilizer. This significantly reduced fuel loads, thereby lowering the risk of large‐scale fires. Meanwhile, the impact of this practice did not alter our measurements, as villagers have been consistently collecting surface litter for several decades.

Logging occurrences and intensities were primarily influenced by the socioeconomic context of each period. Generally, logging activities were identified in the south and west aspects, as well as at the foot of the north aspect, particularly in areas close to settlements, at lower elevations, or with better accessibility (Figure 5). This pattern aligns with global deforestation trends in mountainous regions (Chen et al. 2024). Despite the commercial logging ban implemented in 1998, logging for personal use has continued near villages on the south and west slopes, especially at elevations around 3000 m. Consequently, secondary forests, consisting of either pure *Pinus yunnanensis* or mixed broadleaf species, are now prevalent at these elevations, largely due to a combination of logging and fire disturbances. During the 1980s and early 1990s, commercial logging was widespread, particularly on the southern aspect below 2900 m and along logging trails at 2900–3200 m on the western slope. A large proportion of mature *P. yunnanensis* trees were removed, leading to a conversion toward mixed broadleaf forests dominated by pioneer species, a process similar to species composition changes reported in other studies (Adie et al. 2013; Ngo et al. 2013; Vlam et al. 2016). Logging was also associated with herding activities, as evidenced by the presence of two herder huts at 3500 to 3600 m, with corresponding tree ring records on the west aspect from the 1950s.

Topography, climate, and road construction were the primary drivers of landslides in this forest, which we deduced from our results of disturbance agents and interviews. The steep and unstable terrain in the remote north and east aspects was particularly prone to landslides during the rainy seasons and snowmelt periods (Hasegawa et al. 2008; Biswas and Pal 2016). On the other hand, landslides at the bottom of the south slope since the 1980s were majorly caused by logging and road development activities, which have been reported as deforestation and road construction exacerbating landslide risks (Zhang et al. 2003; Sidle et al. 2010).

Topography was the primary driver of icy precipitation events, with severe occurrences typically happening at elevations between 3500 and 4000 m on the south and west aspects. This phenomenon resulted from the air masses at higher elevations on the warmer sides of the mountain being more susceptible to icy precipitation. Tree mortality was directly caused by mechanical damage from these events (Riley 1953).

In addition, tree regeneration from gaps has been largely used as an indicator of disturbances beside the growth release of tree rings from living trees (Nowacki and Abrams 1997). On the east aspect, gap‐origin *Abies georgei* trees suggested recurrent landslides, particularly in the 1960s, 1980s, and 1990s. Landslides typically uproot large areas of trees, creating open spaces for regeneration when mother trees are nearby. Moreover, gap regeneration of shade‐intolerant species like *Betula utilis* (Niinemets and Valladares 2006) was specifically relevant to past disturbances (Nowacki and Abrams 1997; Vlam et al. 2016), hinting at potential shifts in species composition over time.

### 4.2 Fire Impacts on Carbon

Soil, woody plant, and overall ecosystem carbon were observed to increase with the decades since the last fire (Figures 7, 8). These results aligned with previous studies on post‐fire carbon stocks in living trees, soil, and ecosystems in Mediterranean forests (Kaye et al. 2010), dry sclerophyll forests in Australia (Sawyer et al. 2018), and boreal forests (Seedre et al. 2014; Larjavaara et al. 2017; Palviainen et al. 2020). These consistent findings across diverse forest ecosystems underscore the critical role of fire in shaping long‐term carbon dynamics for two predominant ecosystem components, soil and woody plants.

Post‐fire soil carbon exhibited a faster recovery rate than woody plant carbon in our study area, a pattern also observed in tropical dryland forests of Indonesia (Adinugroho et al. 2022). This accelerated recovery in soil carbon can be attributed to vertical soil nutrient cycling, the decomposition of plant debris, and the activity of soil microbiomes (Attiwill and Adams 1993), as fire predominantly affects the topsoil layer (Pellegrini et al. 2021).

In contrast to woody plants, non‐woody plant carbon showed a decreasing trend with the decades since the last fire. Initially, herbaceous plants dominate after a fire due to their ability to rapidly colonize and take advantage of the increased availability of sunlight, nutrients, and open space created by the fire. However, as time progresses, woody plants gradually re‐establish and grow taller, creating a closed canopy that reduces the availability of sunlight reaching the forest floor. Consequently, the competitive advantage shifts from herbaceous plants to woody plants, leading to a decline in the abundance and carbon storage of non‐woody plants (Arianoutsou‐Faraggitaki 1984).

Since anthropogenic activities influence the spatial and temporal variation of fire occurrences (Han et al. 2015), the observed correlations between fires in the 1950s–1970s and the absence of recent fire showed a positive relationship with soil, woody plants, and overall ecosystem carbon. This suggests that carbon stocks had either recovered after 50 years or the decades since the last fire partially influenced carbon accumulation. Various factors, such as fire intensity, fire frequency, plant type, and topography, concurrently influenced carbon dynamics post‐fire (Pellegrini et al. 2021; Haukenes et al. 2022; Bill et al. 2023). Fires in the 1980s–1990s exhibited the most pronounced negative impacts on woody plants, which could be attributed to more severe fires during this period.

The link between pre‐fire and post‐fire species composition has been established as a significant factor affecting long‐term carbon recovery trends (Han et al. 2015). In our study, most fires occurred in *Pinus yunnanensis*‐dominated forests below 3300 m. This species is serotinous, meaning that high temperatures from fire facilitate the opening of its cones (Su et al. 2015). We observed that forest composition remained unchanged post‐fire, suggesting a stable trajectory of carbon accumulation similar to pre‐fire conditions, consistent with findings in other ecosystems, e.g., Mack et al. (2021).

### 4.3 Logging Impacts on Carbon

Anthropogenic activities can have legacies on forest structure that persist for centuries (Aakala et al. 2023). We found that the negative impacts of logging were most pronounced in woody plant and overall ecosystem carbon densities, while the impacts on soil and litter were comparatively less significant (Figures 7, 8). These results align with observations from various ecosystems globally, e.g., Stas et al. (2020), Seedre et al. (2014). In addition, the carbon recovery rate was higher following logging compared to fire. Logging often allows for quicker regrowth of pioneer and fast‐growing tree species, regardless of whether it alters pre‐logging species composition and forest structure, which can rapidly sequester carbon (Adie et al. 2013; Vlam et al. 2016). Fire, however, can lead to more complex ecological processes and interactions among ecosystem components, potentially resulting in slower plant recovery and regrowth of woody plant species (Goetz et al. 2012; Pellegrini et al. 2021).

The impact of logging on carbon was largely determined by socioeconomic contexts, resulting in variations in intensity, timing, and method (Stas et al. 2020). In this study area, selective logging was the major harvest regime and was species‐specific. From the interviews, we gathered information about the quantity and types of timber utilized by local communities, which indicated the potential cutting amount and locations. Each household required 2.5 tons of wood annually for heating and cooking. Moreover, houses in the area are predominantly made of wood, with *Pinus* spp. commonly used for constructing walls. *Tsuga* spp. or *Pseudotsuga* spp., which typically grow at elevations between 3100 and 3500 m, were used for the two crucial pillars inside houses that bear most of the structural weight. Additionally, *Abies* spp. and *Picea* spp., which grow at higher elevations, were utilized for constructing roofs.

Besides logging, other anthropogenic activities, such as collecting medical herbaceous plants for sale, gathering woody debris for firewood, and collecting litter, also affected carbon densities, although the impact was minimal. Additionally, the tendency to prioritize deadwood collection near settlements, where logging was frequently repeated, contributed to the observed negative correlation between logging and woody debris carbon density in our results. Villagers collected litter, especially pine needles, for pigsties or stables during winter. These were then either used as fertilizer or as fuel for heating, further contributing to the reduction in litter carbon.

Logging intensity in our study area increased after 1950, gaining momentum in the late 1960s (Hillman 2003; Willson 2006). Large‐diameter trees were harvested for industrial purposes in locations with higher carbon stocks.

Woody plant carbon in plots logged in the 1980s and 1990s was lower than in plots without recent logging. During this period, improved infrastructure, such as upgraded roads and trails, made timber transport more accessible and cost‐effective, facilitating the intensification of logging activities and leading to the removal of medium‐ to large‐sized trees. In general, annual harvests in Yunnan province were more than three times the amount prescribed under the State Plan, even excluding fuelwood use and forest clearance for agriculture (Colchester 2004).

Since the 2000s, the negative impact of logging on woody plant carbon has persisted despite the commercial logging ban. This was largely because the locations chosen for harvesting were typically close to the villages. In these areas, which had already experienced frequent and intensive logging or fire, carbon stocks were generally low due to cumulative disturbances. During this period, in response to the commercial logging ban in 1998, villagers harvested timber only for their own use. Therefore, the intensity of such harvesting has been much lower than in previous decades due to restrictions on construction wood and firewood harvesting imposed by local forest authorities.

### 4.4 Topography Impacts on Carbon

Topography not only influenced fire (Margolis et al. 2022), landslides, icy precipitations, and logging regimes but also significantly altered carbon distribution. In this study, even after accounting for the impacts of fires and logging in the model, both woody plants and overall ecosystem carbon densities consistently exhibited a hump‐shaped pattern (Figure 10). This finding aligns with previous research on aboveground plant biomass along elevational gradients in this area (Cheng et al. 2023), where disturbance variables were not considered. The distribution pattern observed can be attributed to the hot‐dry valley climate at lower elevations and the cold climate at higher elevations, which are primary driving factors influencing plant structure and carbon sequestration (McCain and Grytnes 2010). Furthermore, disturbances are more prevalent at lower elevations, resulting in reduced carbon levels in these areas (Han et al. 2015).

The trends in carbon densities along elevational gradients showed a significant shift when accounting for the effects of time of last fire or logging, as well as their combined impact. This suggests that both the legacy effects of past disturbances and their cumulative influence over time significantly modulate carbon storage across elevations (Swetnam et al. 2017; Turner and Seidl 2023). These findings help us understand how human and natural processes interact over time and show why land management in fire‐prone areas must consider both elevation and disturbance history. Additionally, elevation‐specific factors such as temperature, precipitation, soil type, and species composition shape how ecosystems recover from disturbances, leading to diverse post‐disturbance regeneration dynamics and succession processes (Wolf et al. 2021).

The carbon density of woody debris demonstrated a positive correlation with site moisture levels. This finding aligns with a study conducted in the temperate forests of central Poland, which suggested that high site moisture may have impeded the decomposition process of organic matter (Piaszczyk et al. 2022). Similarly, areas with higher moisture levels exhibited slightly higher, though nonstatistically significant, soil carbon levels. Similar trends have been observed in mountainous regions in Bavaria, southeastern Germany (Wiesmeier et al. 2014), southern Spain (Román‐Sánchez et al. 2018), Colorado, USA (Swetnam et al. 2017), and Norway (Haukenes et al. 2022). This could be the result of enhanced soil microbial activity and decomposition processes (Attiwill and Adams 1993; Bill et al. 2023). The distinct responses of woody debris and soil carbon to varying site moisture levels elucidated additional factors driving carbon cycling under diverse moisture conditions.

### 4.5 Limitations

In this study, we did not estimate fire and logging intensities, although they likely varied among the sampling plots and could have significantly influenced carbon dynamics alongside disturbance timing. We adopted a conservative approach to identifying disturbance agents since 1950, including only those disturbances detected through fire scars, satellite images, and historical documents. It is possible that additional disturbances may have occurred in the study area that eluded capture by these sources. For example, linking reported disturbances from interviews to specific plots proved difficult; we only included those events for which we had high confidence, which may have resulted in the exclusion of some disturbances that were not definitively identified. Additionally, leaf litter collection by villagers may have influenced carbon accumulation, though such impacts are not accounted for in our models.

## 5 Conclusion

Our study aimed to disentangle the effects of multiple disturbances on different ecosystem carbon components in a human‐impacted mountainous forest in northwestern Yunnan, China. Our findings demonstrate the benefits of using different types of data together, including tree rings, fire scars, satellite imagery, historical documents, interviews, and field measurements on forest structure, especially in areas where official records are limited or missing. This combined approach helps us to successfully ascertain disturbance agents since 1950 and analyze their effects on carbon storage and allows us to consider the direct impacts of fires, logging, and other disturbances, as well as the roles played by topography and proximity to settlements.

We also found that both disturbance agents and timing, along with elevation and how close the forest is to villages, have long‐term effects on how much carbon forests store. This leads to practical implications for both forest carbon inventory and policies to manage disturbances while mitigating climate change.

Though our study only focuses on one mountain forest in northwestern Yunnan province, it broadly shows how human activity and natural processes shape forest carbon together over time. Bringing together local socioeconomic and natural disturbance history can help us manage forests for carbon sequestration and make decisions that are aligned with local conditions.

## Author Contributions

**Zhongqian Cheng:** conceptualization (equal), data curation (lead), formal analysis (lead), funding acquisition (equal), investigation (lead), methodology (lead), project administration (equal), resources (equal), validation (equal), visualization (lead), writing – original draft (lead), writing – review and editing (equal). **Tuomas Aakala:** formal analysis (supporting), methodology (supporting), supervision (lead), validation (equal), writing – review and editing (equal). **Chengjun Ji:** supervision (equal), writing – review and editing (equal). **Markku Larjavaara:** conceptualization (equal), funding acquisition (equal), investigation (equal), project administration (equal), supervision (lead), validation (equal), writing – review and editing (equal).

## Conflicts of Interest

The authors declare no conflicts of interest.

## Supporting information


**Figure S1:** ece372165‐sup‐0001‐Supporting information.docx.

**Data S1:** ece372165‐sup‐0002‐Original data.xlsx.

## Data Availability

The data sets supporting this article have been uploaded as part of the Supporting Information.
