# Supplementary material for: Disturbance Dynamics and Its Effects on Carbon in Human‐Impacted Mountain Forests in Northwestern Yunnan, China
Source: Ecol Evol. 2025 Sep 14;15(9):e72165. doi: 10.1002/ece3.72165 (PMC12434075; doi:10.1002/ece3.72165)
Supplement: Supplementary file 1 — Figure S1: ece372165‐sup‐0001‐Supporting information.docx. [file ECE3-15-e72165-s002.docx]

Disturbance dynamics and its effects on carbon in human-impacted mountain forests in northwestern Yunnan, China

Zhongqian Cheng^1,2*^, Tuomas Aakala^3^, Chengjun Ji^1*^, Markku Larjavaara^4^

*1 Institute of Ecology and Key Laboratory for Earth Surface Processes of the Ministry of Education, College of Urban and Environmental Sciences, Peking University, 100871 Beijing, China*

*2 Arthur Temple College of Forestry and Agriculture, Stephen F. Austin State University, Nacogdoches, 75962 Texas, USA.*

*3 School of Forest Sciences, University of Eastern Finland, P. O. Box 111, 80101 Joensuu, Finland*

*4 Department of Forest Sciences, University of Helsinki, P.O. Box 27, 00014 Helsinki, Finland*

*ZC: 0000-0002-9846-0747; TA: 0000-0003-0160-6410; ML: 0000-0002-3484-889X*

Corresponding authors: Zhongqian Cheng: [chengz@sfasu.edu](mailto:chengz@sfasu.edu); Chengjun Ji: jicj@pku.edu.cn


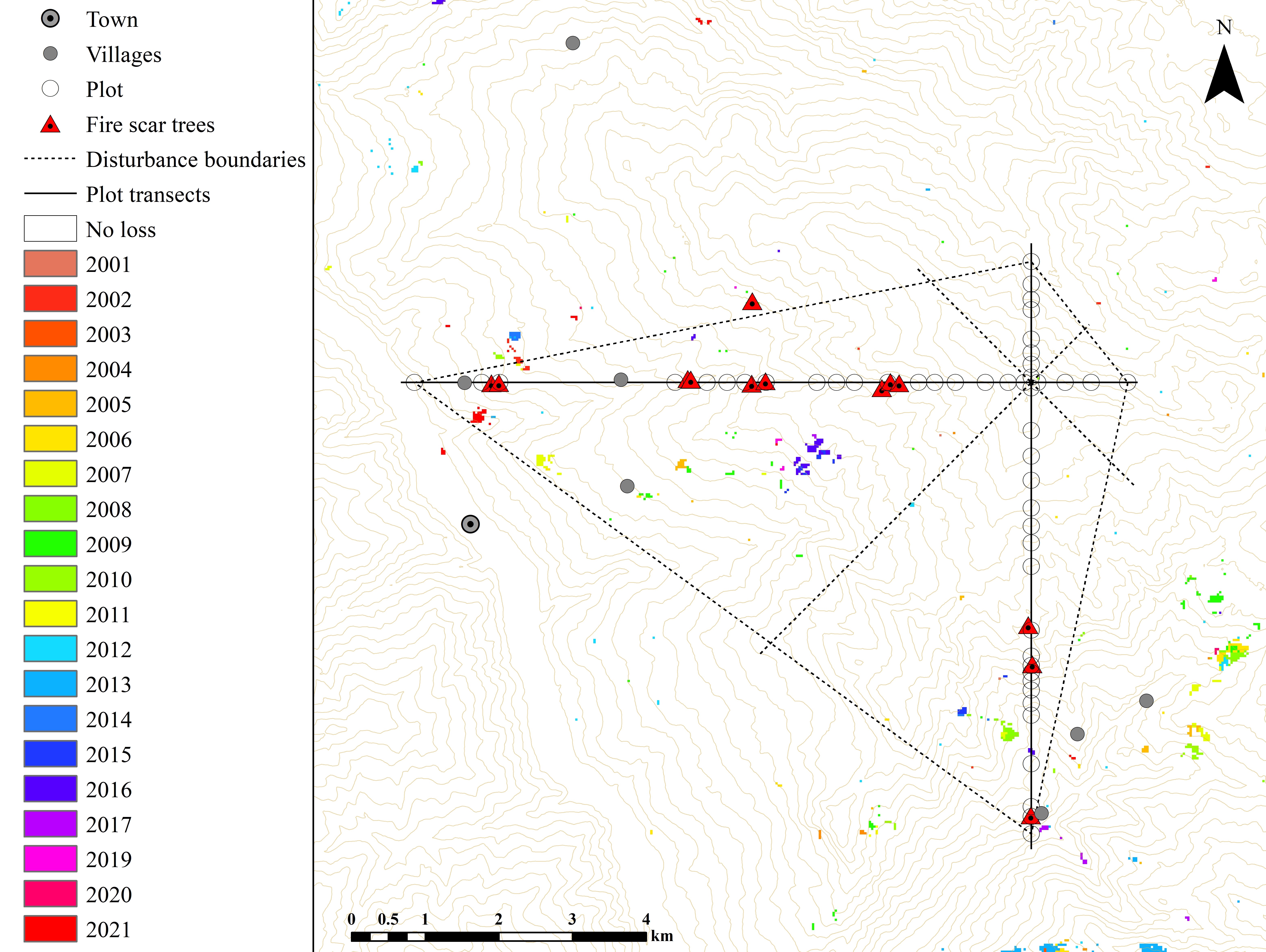


Figure S1. Fire scar samples, transect used for grouping forest loss, and pooled annual fire (Tyukavina *et al.*, 2022) and non-fire (Hansen *et al.*, 2013) loss. Open circles are the plots. Red triangles with dot are the location for fire scared trees. Solid lines are the transects (aspects) for locating plot. Dashed lines are the transects for setting disturbance boundaries.


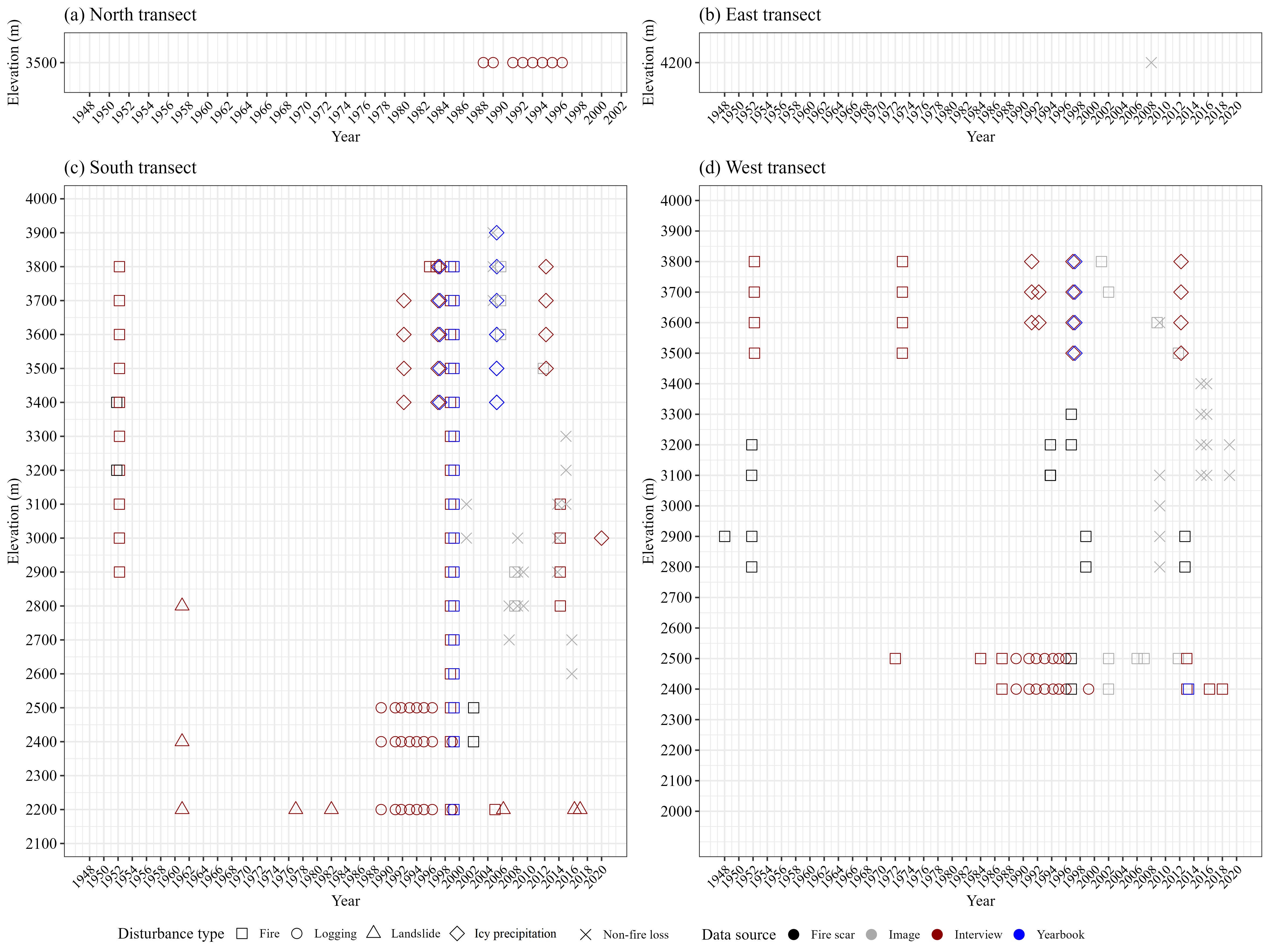


Figure S2. Summary of disturbances since 1948 based on fire scars, satellite-based map (Hansen *et al.*, 2013; Tyukavina *et al.*, 2022), interviews, and yearbooks.


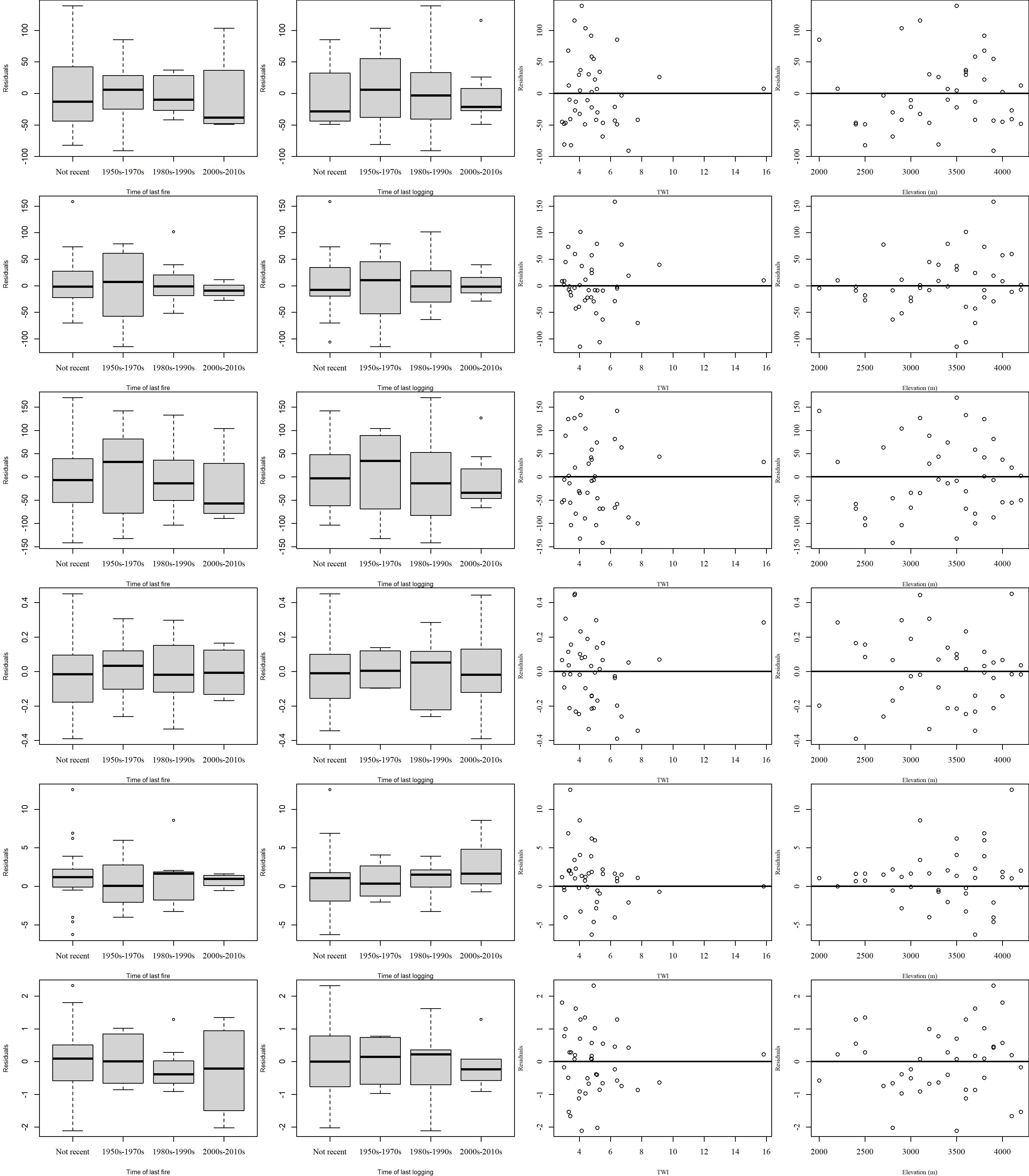


Figure S3. residuals plot of GAM models.

# References

Hansen M.C., Potapov P.V., Moore R.*, et al.*, 2013. High-resolution global maps of 21st-century forest cover change. Science 342, 850-853. 10.1126/science.1244693

Tyukavina A., Potapov P., Hansen M.C.*, et al.*, 2022. Global trends of forest loss due to fire from 2001 to 2019. Frontiers in Remote Sensing 3, 825190. 10.3389/frsen.2022.825190
